# Sustainable Diesel from Rapeseed Oil Esters by Sequential Semi‐Hydrogenation, Double Bond Isomerization, and Metathesis

**DOI:** 10.1002/chem.202500523

**Published:** 2025-03-11

**Authors:** Mykhailo Kondratiuk, Maximilian L. Spiekermann, Thomas Seidensticker, Lukas J. Gooßen

**Affiliations:** ^1^ Evonik Chair of Organic Chemistry Ruhr-Universität Bochum Universitätsstr. 150 44801 Bochum Germany; ^2^ Department for Biochemical and Chemical Engineering Laboratory for Industrial Chemistry TU Dortmund University Emil-Figge-Str. 66 44265 Dortmund Germany

**Keywords:** biofuels, selective partial hydrogenation, oleate, isomerization, ethenolysis

## Abstract

Rapeseed oil methyl esters (RME) have been converted to biofuel with a boiling point curve that fulfills the EN 590 specifications for modern diesel engines using a robust, three‐step process. In the first step, the polyunsaturated esters of the RME were semi‐hydrogenated in the presence of 20 ppm of a solvent‐stabilized Pd^0^ colloid. The resulting mono‐unsaturated fatty esters were further converted into a defined mixture of double‐bond isomers by passing them over inexpensive, Brønsted‐acidic Amberlyst 15 resin at high space‐time yields (1.3 kg⋅L^−1^⋅h^−1^). The resulting mixture was then converted into a blend of terminally unsaturated olefins and monoesters, with <4.9 % diesters and <21 % saturated fatty esters by cross‐metathesis with technical‐grade ethylene. In this step, 50 ppm of a cyclic alkyl amino carbene (CAAC) Ru catalyst M1001 was used to achieve record‐setting conversions (91 %) and selectivities (94 %). All three steps were conducted with neat feedstock at mild temperatures (60–100 °C). This demonstrates that sustainable diesel fuel for use in contemporary diesel engines is accessible from RME and ethylene via a short set of industrially viable reaction steps.

## Graphical abstract

This article describes a robust, sustainable, and industrially viable three‐step process to transform rapeseed oil methyl esters (RME) into biofuel with a boiling point curve that fulfills the modern diesel specifications for the use in contemporary diesel engines. This process consists of selective partial hydrogenation of RME, followed by heterogeneously‐catalyzed double bond isomerization and cross‐metathesis with ethylene.

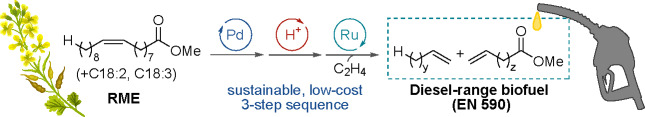

## Introduction

Stabilizing the rising global temperatures requires a substantial reduction in emissions of anthropogenic carbon dioxide.[^1^, ^2^] However, certain sectors, such as aviation, heavy trucking, and maritime transportation, present substantial challenges to decarbonization.^[3]^ Currently, global transportation depends almost entirely (>90 %) on combustion fuels, mostly diesel. Diesel fuel is favored for its high energy density, low cost, the extensive supply infrastructure, and its use in robust engines.[^4^, ^5^]

**Figure 1:**
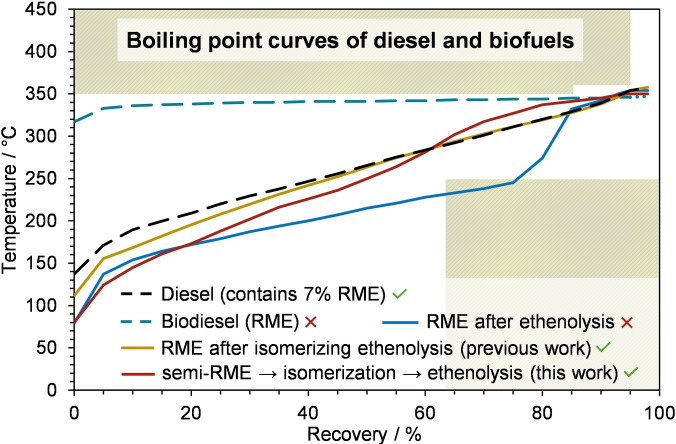
Boiling point curves of commercial diesel and biodiesel, as well as RME modified by ethenolysis, by isomerizing ethenolysis,^[9]^ and by sequential semi‐hydrogenation‐isomerization‐ethenolysis presented in this work.

From an ecological perspective, it is desirable to maximize the proportion of renewable components in diesel fuel, but from a practical standpoint, the resulting fuel mixture should be suitable for the use in state‐of‐the‐art diesel engines without major modifications or expensive additives. A critical factor for good engine performance, especially at startup,^[6]^ is to fulfill the boiling behavior specified in EN 590. Fossil diesel is a complex mixture of linear and branched hydrocarbons with 9–22 carbon atoms, resulting in an evenly rising boiling point curve ranging from 150 to 360 °C. In contrast, biodiesel mostly consists of five fatty acid esters with chain lengths of 16 or 18 carbon atoms.^[7]^ This results in a narrow boiling point curve outside the specified limitations (Figure 1). No more than 7 vol‐% biodiesel can be added to fulfill EN 590.^[8]^

Hydrotreated Vegetable Oils (HVOs)[^10^, ^11^] are generated from fatty acid derivatives by hydrogenation and partial skeletal isomerization. In contrast to traditional biodiesel, HVO furnishes a boiling point curve within the EN590 limits but it still does not match that of fossil diesel; moreover, the density of HVO is lower than the specifications demand.[^12^, ^13^, ^14^] Although an engine can be rendered compatible with 100 %‐HVO through some adjustments, this feature is currently present in a relatively small fraction of newer vehicles.^[15]^ The highest content of renewables in a commercial fuel that fulfills EN 590 specification is reached in the R‐33 diesel, which consists of 7 vol‐% biodiesel, 26 vol‐% HVO, and 67 vol‐% fossil diesel.^[16]^ A simple way to reduce the boiling point of RME seems to be its cross‐metathesis of RME with ethylene (ethenolysis) to give mainly 1‐decene and methyl‐dec‐9‐enoate. However, the boiling point of the latter dominates the curve over a too broad range from 40 % to 75 % recovery, which shifts it outside the EN 590 specification (Figure 1).

The Gooßen group demonstrated that the EN590 specifications can be fulfilled by subjecting rapeseed methyl esters (RME) to isomerizing metathesis (ISOMET) in the presence of ethylene. The simultaneous, continuous, and orthogonal action of an isomerization catalyst, a cross‐metathesis catalyst, and an ethenolysis catalyst on a 1 : 1 mixture of RME and ethylene was tuned in a way that it yields a blend of internal olefins, mono and diesters with a boiling point curve that meets EN 590 specifications (Figure 1).[^9^, ^17^]

In the past 5 years, key contributions from Hartwig, Scott, Guironnet, Tuba, and Peters research groups have further developed ISOMET into useful tool for upcycling of non‐degradable polyolefins.[^18^, ^19^, ^20^, ^21^, ^22^, ^23^, ^24^, ^25^] However, ISOMET is a delicate tandem process based on expensive homogeneous catalysts in rather high loadings,[^26^, ^27^] which precludes its application in commercial fuel synthesis. For instance, the isomerizing ethenolysis of RME calls for two Hoveyda‐Grubbs‐type Ru catalysts (one with an N‐heterocyclic carbene (NHC), one with a cyclic alkyl amino carbene ligand (CAAC), 1000 ppm each) and a Pd‐based isomerization catalyst (4000 ppm).^[9]^

**Scheme 1:**
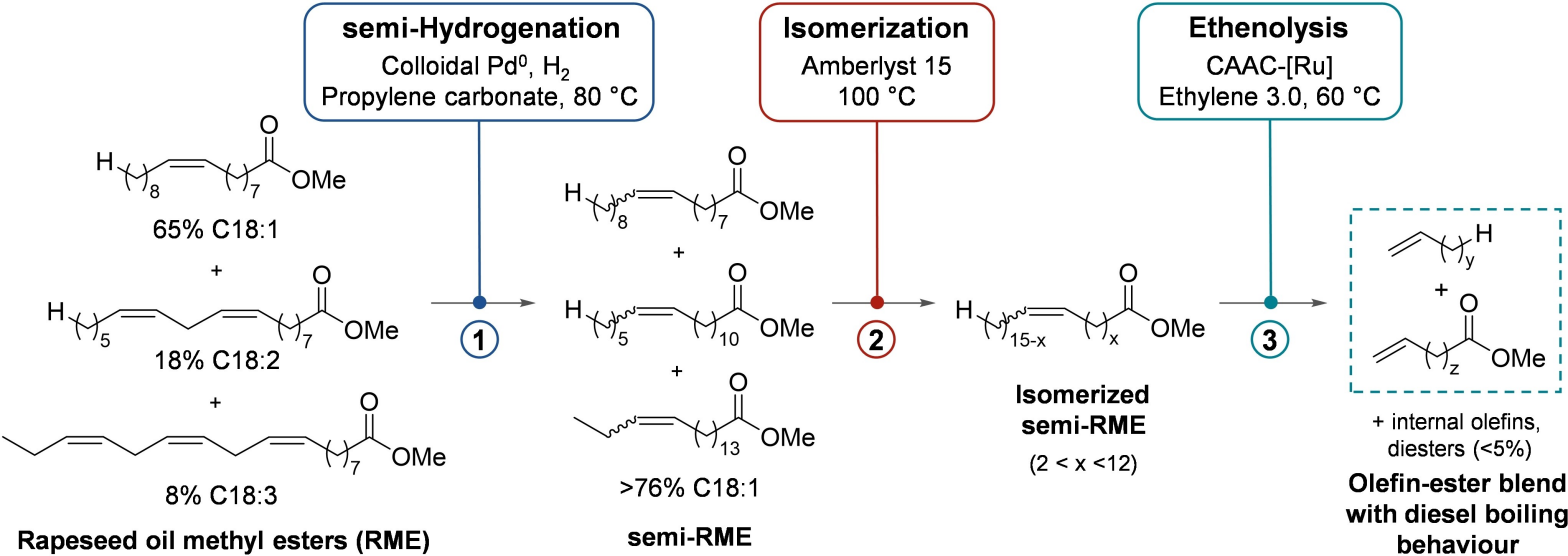
In three steps from RME and ethylene to a blend of terminally unsaturated esters and olefins with diesel‐like boiling point curve.

We now sought for a more straightforward, less expensive way to tailor the boiling point curve of fatty acid mixtures. We reasoned that, by moving the double bond of methyl oleate away from its original position by only 2–3 carbon atoms on average, a sufficiently broad distribution of double bond isomers would form. A subsequent ethenolysis step would generate a blend of terminal olefins and terminally unsaturated monoesters (Scheme 1). The advantage of this approach over ISOMET is that both steps can be performed separately, each under individually optimized conditions. The isomerization might even be conducted over an inexpensive, heterogeneous catalyst. The boiling point curve of the multicomponent mixture generated in the ethenolysis step should be as smoothly raising as that of petrodiesel, and its shape could be tuned by adjusting the average number of double bond migration steps.

The number of isomerization steps is critical in this approach. If a too high number of isomerization steps is allowed, the double bond migrates towards the alkyl ends of the chain, so that unwanted low‐boiling products form. It will also move close to the carboxylate group, leading to irreversible formation of relatively high‐boiling γ‐stearolactone. The latter will form as the sole product, if unsaturated fatty acids or esters are allowed to react with a highly active isomerization catalyst over prolonged time.^[28]^

**Figure 2:**
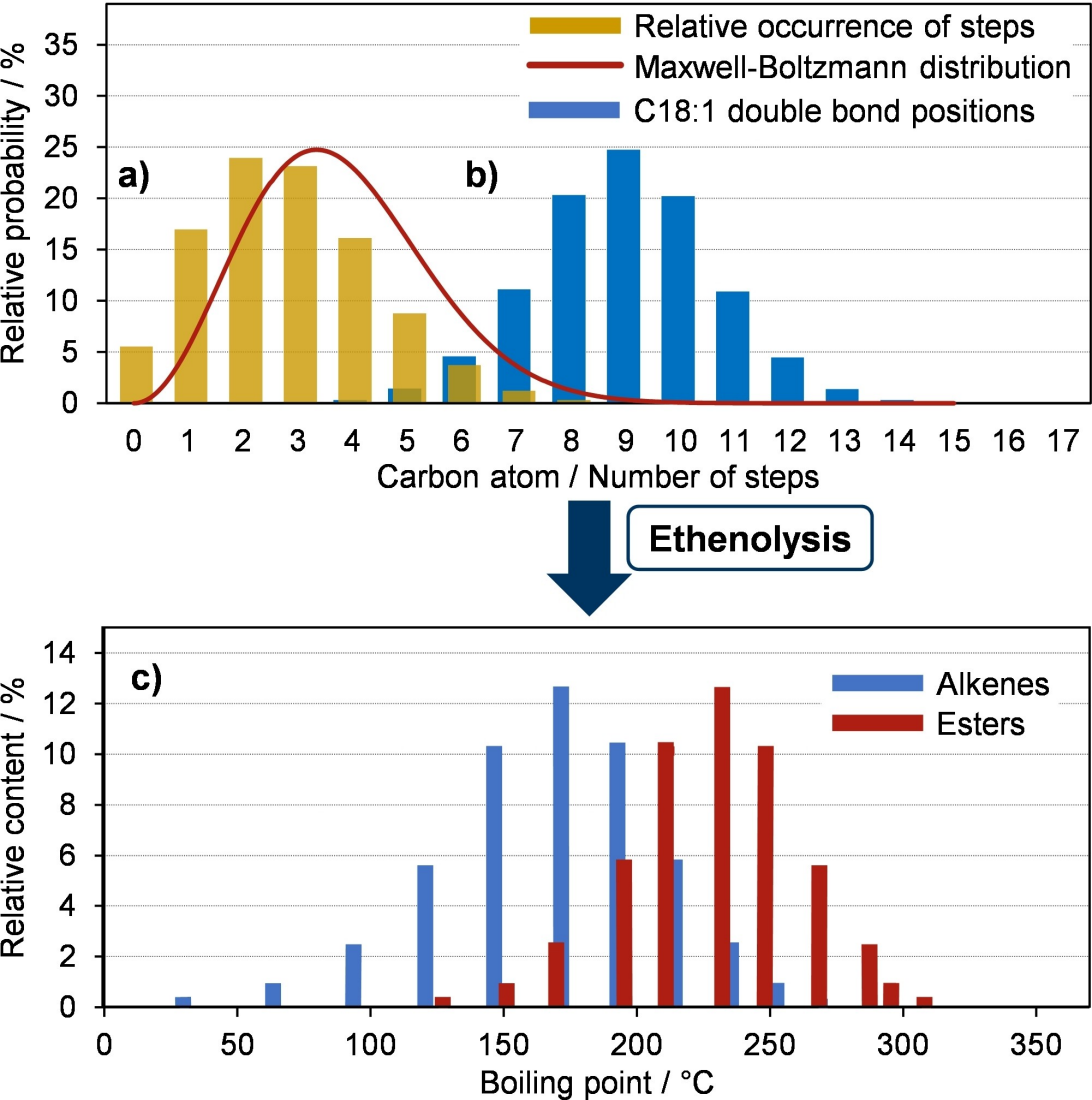
a) Maxwell‐Boltzmann distribution of double bond migration steps calculated around an average of 3.3 steps; b) Simulated distribution of double bond isomers when subjecting C18 : 1 cis/trans‐9 to this distribution of isomerization steps; c) Expected ethenolysis products and their boiling points.^[29]^

The effect of the average number of isomerization steps on the boiling points of ethenolyzed unsaturated FAMEs is visualized in Figure 2. We reasoned that the number of isomerization steps should follow a Maxwell‐Boltzmann distribution, if methyl oleate is passed through a heterogeneous isomerization catalyst. We set the ratio of molecules with sufficient energy to migrate over >6 steps to 1 %, thus restricting the amount of stearolactone, butene, propene and ethylene to trace quantities. The Maxwell‐Boltzmann distribution, calculated with this boundary, results in a distribution of double bond migration steps around an average of 3.3 steps (Figure 2a). In an ensemble of 100000 methyl oleate molecules (double‐bond in 9,10‐position), the double bond was allowed to migrate over a number of steps which was defined by the Maxwell‐Boltzmann distribution probabilities, each time with the same probability in both directions. The resulting distribution of double bond isomers is shown in Figure 2b. Expectedly, isomers with the double bond in 9‐position – same as in the starting material – are the most likely isomers in this distribution. The probability of other positional isomers thus gradually declines with increasing distance from this position, reaching <0.1 % for positions that are >5 carbons away. Ethenolysis of this mixture would lead to a blend of products with a distribution of boiling points similar to that of the petrodiesel components (Figure 2c). On average, their boiling point is somewhat lower than that of the diesel components, which is advantageous since crude FAME mixtures inevitably contain saturated FAMEs which remain unchanged over the course of the isomerization and ethenolysis and thus increase the proportion of high boiling materials.

However, if one wants to come close to this targeted product distribution (Figure 2b), the ethenolysis would have to set new records with regard to conversion and selectivity. Incomplete conversion would lead to a high content of C18 esters in the product, low selectivity in the ethenolysis would give rise to high boiling diesters as byproducts. Significant quantities of both components would shift the right side of boiling point curve out of specifications. Reaching high conversions poses a formidable challenge, since in the literature, high TON numbers for the ethenolysis of methyl oleate have so far been achieved mostly at moderate conversions.[^30^, ^31^, ^32^, ^33^]

Mecking and co‐workers demonstrated that a one‐pot, three‐step ethenolysis‐isomerization‐ethenolysis sequence is suitable to convert fatty acid methyl esters (FAMEs) into a reasonably well‐distributed blend of C_3_–C_10_ alkenes and esters. However, they used relatively high loadings of homogeneous catalysts for all three steps, i. e. a total of 4600 ppm UltraCat (UC) and 4000 ppm [(dtpbx)Pd(OTf)_2_].^[34]^ In the search for a heterogenous catalyst that promotes selective double bond migration of RME, we have recently investigated H‐mordenite and other zeolite catalysts.^[35]^ However, at reaction temperatures of 290–300 °C, the methyl linoleate (C18 : 2, 18 %) and linolenate (C18 : 3, 8 %) content caused the formation of >16 % non‐volatile byproducts via Diels‐Alder reactions and double bond polymerizations.^[35]^ These examples show that the content of multiply unsaturated fatty acids is a major problem when trying to generate biodiesel for conventional engines via an isomerization/ethenolysis sequence. We have now addressed this issue by upfront converting the multiply unsaturated fatty esters of RME into mono‐unsaturated derivatives using a catalytic semi‐hydrogenation that was developed by Behr and Seidensticker (Scheme 1).[^36^, ^37^] The double bonds were then migrated along the carbon chain by passing them through a tube reactor filled with Amberlyst 15 resin at only 100 °C yielding fatty esters with a broad distribution of double bond isomers and only <2 % non‐volatile byproducts. The carbon chains of this ester blend were then shortened by a high‐yielding ethenolysis step (84 % conversion) using only 50 ppm of a CAAC‐ruthenium catalyst (Umicore M1001).[^30^, ^38^] The resulting olefin‐ester blend has its boiling properties compliant with the EN 590 norm^[39]^ (Figure 1).

## Results and Discussion

### Semi‐Hydrogenation and Double Bond Isomerization of Fatty Acid Esters

We started our investigations by probing the effect of multiply‐unsaturated fatty acids on heterogeneously catalyzed double‐bond isomerization. In order to obtain affordable FAME mixtures with no polyunsaturated fatty acids, we semi‐hydrogenated FAME mixtures form natural source following the procedure by Behr et al.^[36]^ A propylene carbonate solution of solvent‐stabilized Pd nanoparticles with a diameter of ca. 4 nm was used as the catalyst. It was generated by heating a solution of Pd(OAc)_2_ in propylene carbonate.^[37]^ A 4 : 1 mixture of FAME with propylene carbonate containing 20 ppm Pd (relative to the molar amount of the used FAME) was hydrogenated at 80 °C for 2 h at 10 bar hydrogen pressure. Upon cooling, the liquid phases separated. The semi‐hydrogenated FAME (the upper phase) was analyzed by GC and purified by distillation, the lower phase with the catalyst was recycled. The Pd‐content of the product was below the detection limit of our instruments (<1 ppm). It has previously been shown that the catalyst phase can be reused several times without loss in activity.^[36]^

For our investigations, high oleate sunflower oil methyl ester (HOSOME) was semi‐hydrogenated to “semi‐HOSOME”, and RME – to “semi‐RME” over 20 ppm colloidal palladium at 10 bar hydrogen and 80 °C for 2 h. The used HOSOME contained 87 % methyl oleate, 6 % linoleate, 7 % saturated, and no triple‐unsaturated esters, as determined upon GC analysis on a J&W DB‐FastFAME column. Semi‐hydrogenation resulted in >90 % of C18 : 1 esters with full elimination of linoleate. Moreover, it caused some double bond migration on the C18 : 1 scaffold over one carbon atom. To determine the exact ratios of the positional isomers in semi‐HOSOME, we subjected it to ozonolysis‐reduction and analyzed the resulting fragments of aldehydes and oxoesters. Based on that, semi‐HOSOME consisted of 82 % C18 : 1 *cis/trans‐9*, <4 % each of *cis/trans‐8* and *−10*, <2.5 % total of *cis/trans‐11* and *−12*, and 9 % saturated fatty esters.

In contrast to HOSOME, RME contains only 65 % oleate, along with 20 % linoleate, 9 % linolenate, and 6 % saturated esters. In order to ensure full removal of C18 : 2 and C18 : 3 from the feedstock, its semi‐hydrogenation was performed under somewhat forcing conditions. This indeed afforded full removal of polyunsaturated FAMEs, but at the price of an increased methyl stearate content (19 %). After 2 h reaction time, the resulting “semi‐RME” contained >76 % C18 : 1 compounds (42 % C18 : 1 *cis/trans‐9*, 10–11 % each of *cis/trans‐8* and *−10*, 6–7 each of *cis/trans‐11* and *−12*) along with 23 % saturated esters. A flow‐through setup with constant reaction monitoring should allow to reach higher selectivities for such technical‐grade substrates with varying purity.

Due to its higher similarity to pure methyl oleate, we used semi‐HOSOME rather than semi‐RME to systematically investigate the influence of multiply unsaturated fatty esters on the heterogeneously catalyzed double bond isomerization. With the help of an HPLC pump, the substrate was passed at a flow rate of 0.1 mL/min through a 280 mm‐long tube reactor with a 10 mm inner diameter, filled with 0.5 g H‐mordenite and 9.5 g inert carrier (SiC) heated to 290 °C.^[35]^ The yields of linear, branched, and oligomeric products as well as of γ‐stearolactone were determined by hydrogenating the product mixture over Pd/C and then performing GC analysis. The methyl palmitate content of the starting material was utilized as a standard against which the other components of the hydrogenated product mixture were quantified. The difference in the mass balance between the product chromatogram and the starting material chromatogram was used to calculate the content of non‐volatile products. To determine the positional distribution of the double bonds in the product mixtures, aliquots of the latter were subjected to ozonolysis‐reduction, and the signals of the resulting mixture of aldehydes and oxoesters were integrated and assigned to the corresponding linear C18 : 1 products.^[35]^

When converting semi‐HOSOME under these conditions, 70 % of linear fatty ester isomers were obtained along with 18 % branched isomers and 12 % of non‐volatile products. In sharp contrast, only 57 % of linear isomers were obtained when applying the optimized conditions to the isomerization of non‐modified RME. As much as 19 % branched isomers and 24 % non‐volatile byproducts were formed (see Supporting Information, Figure S4). This finding illustrates how sharply the efficiency of the isomerization reaction increases when linoleate and linolenate are removed from the substrate.

**Figure 3:**
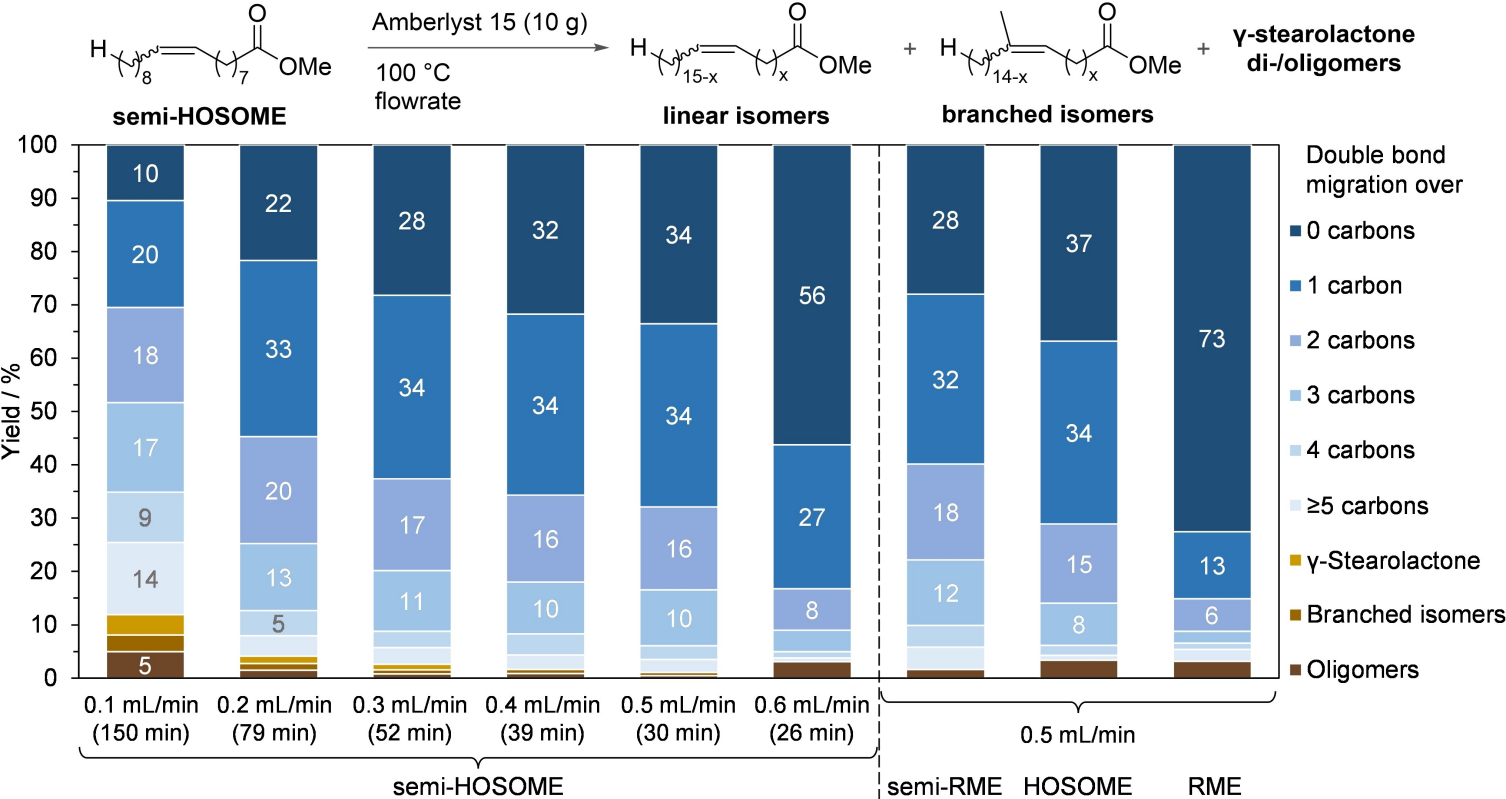
Results of parameter and substrate screening for the isomerization step using Amberlyst 15 in the flow reactor. Conditions: 10 g Amberlyst 15 dry, 100 °C. X‐axis values in brackets: mean residence time at the set flowrate. Stacked column blocks with yields below 5 % are not labelled due to space restrictions. The distribution of double bond positions was quantified using ozonolysis. The reactor was equilibrated for three mean residence times upon collecting the sample aliquots.

To address the problem of the high reaction temperatures, we screened various acidic catalysts at a temperature of only 120 °C and a flow rate of 0.1 mL/min. In contrast to zeolites, some Brønsted acidic ion exchange resins proved to be active even at this temperature. Among six catalysts from the three ion‐exchange resin families (Dowex 50 W, Amberlite, and Amberlyst), the non‐swelling macroreticular resin Amberlyst 15 showed the highest isomerization activity (see Supporting Information, Figure S4).^[40]^ We attribute this to its high cross‐linker content of 20 % and its large pore size of 0.40 mL⋅g^−1^,which allows the large substrate molecules to access its Brønsted‐acidic sites. Isomerization of semi‐HOSOME over 10 g Amberlyst 15 at 100 °C and 0.1 mL/min afforded a near‐equilibrium distribution of linear isomers along with only 5 % oligomers, 3 % branched isomers, and 4 % lactone (Figure 3).^[40]^

We next optimized the flow rate to increase the overall reactor productivity and minimize side product formation. Our target was to get as close as possible to the above‐mentioned product distribution with an average of 3–4 bond migration steps and <1 % migrations over more than 6 bonds. Since isomerization is an equilibrium process, it is not easy to quantify the reaction conversion and yield in a conventional way. After all, the C18 : 1 *cis/trans‐9* isomer is not only the starting material but also the most likely product of multiple double‐bond migrations. This is why the experimental distributions deviate from the idealized distribution shown in Figure 2. After all, even in semi‐HOSOME, only 90 % of the starting material actually are C18 : 1 *cis/trans‐9* esters.

We isomerized semi‐HOSOME at different flowrates (Amberlyst 15 at 100 °C). With an increasing flow rate of the isomerization reaction (and thereby a decreasing average number of isomerization steps), the content of C18 : 1 *cis/trans‐9* isomer in the product increases, whereas that of unwanted byproducts decreases (Figure 3). At 0.5 mL/min, which corresponds to an industrially viable reactor space‐time yield of 1.3 kg⋅L^−1^⋅h^−1^, linear esters are formed in 99 % yield. Under these conditions, 63 % of the double bonds were shifted by more than one position, with total migration over no more than 5 carbon atoms (migration over more than 5 atoms in <1 % total). Non‐volatile products and branched derivatives were formed only in trace amounts (<1 % each) and γ‐stearolactone was not detected. At higher flow rates (≥0.6 mL/min), the conversion dropped markedly. Under the optimal conditions (10 g Amberlyst 15, 100 °C, 0.5 mL/min), not only semi‐HOSOME but also semi‐RME was successfully converted, yielding >98 % linear fatty esters along with <2 % non‐volatile byproducts. This is an important finding since the use of cheap and locally available RME as a feedstock is advantageous from an economic standpoint. The double bond distribution is somewhat different from that observed for semi‐HOSOME, which was to be expected since semi‐RME has a different distribution of double‐bond isomers from the start.

Comparative experiments with HOSOME (6 % C18 : 2) and RME (20 % C18 : 2, 9 % C18 : 3) confirmed that multiply unsaturated fatty acid esters significantly reduce the catalytic activity of the Amberlyst 15 isomerization catalyst. For both substrates, visible amounts of unwanted non‐volatile byproducts were formed (3 %).

Moreover, for RME, as much as 73 % of the product mixture consisted of unreacted material (not only C18 : 1 *cis/trans‐9* but also C18 : 2 and C18 : 3 with a double bond located between carbons 9 and 10). This suggests that multiply unsaturated FAMEs undergo side reactions that block the active sites of Amberlyst 15. It is known that they react with remaining cross‐linker (divinylbenzene) with the formation of covalent bonds. This blocks the access of the substrate to the Brønsted acidic centers.^[41]^

This again illustrates the advantages of the upfront semi‐hydrogenation step on the efficiency of the overall process. The initial high content of polyunsaturated FAMEs in RME vs HOSOME is actually advantageous, because the semi‐hydrogenation also leads to isomers with the double bond on carbons 8, 10, 11, and 12, leading to a broader distribution of isomers after the same amount of isomerization steps.

**Figure 4:**
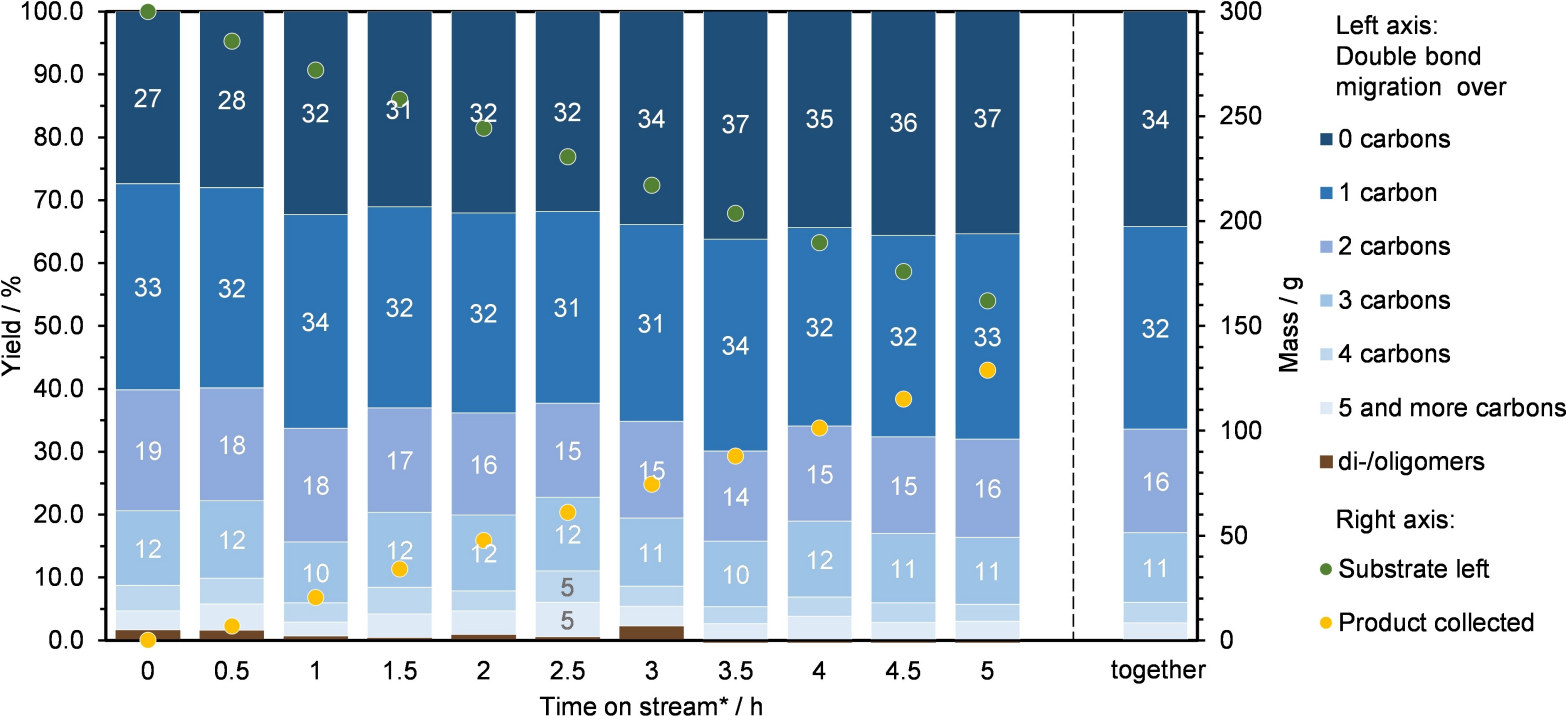
Results of a long‐term isomerization experiment. Conditions: semi‐RME, 10 g Amberlyst 15 dry, 100 °C, 0.5 mL/min. Resulting reactor space‐time yield: 1.33 kg⋅L^−1^⋅h^−1^. *after equilibration for 3 mean residence times.

Having thus found an effective catalyst system for the isomerization of semi‐RME, we moved on to conduct the reaction on a preparative scale. Within five hours of continuous operation at 0.5 ml/min, 150 mL semi‐RME was successfully converted. The reaction yielded linear fatty ester isomers in almost exclusive selectivity. Only traces of non‐volatile byproducts (<1 %) were left behind in a subsequent distillation of the combined product fractions (Figure 4). The reactivity of the Amberlyst 15 slowly decreased over the five‐hour reaction time, so that the content of non‐migrated isomers gradually increased from 27 % to 37 %.

### FAME Ethenolysis and Boiling Point Curves

The preparative isomerization experiment supplied us with sufficient material to move on to the final step of the reaction sequence, the catalytic ethenolysis. Based on our previous findings, we were confident that ethenolysis of the isomerized semi‐RME would deliver low‐boiling product mixtures.^[9]^ Methyl oleate ethenolysis has been studied extensively,[^42^, ^43^, ^44^, ^45^, ^46^] and high TON have been reported, albeit at low conversions.[^30^, ^31^, ^32^, ^33^] Since we were aiming at a product mixture that fulfills the EN 590 boiling specifications, we had to achieve a high conversion of the unsaturated fatty esters with a near‐quantitative selectivity to minimize the content of high‐boiling self‐metathesis products, which can have chain lengths of up to 34 carbon atoms. Even small amounts of high‐boiling byproducts can cause the product mixture to fail to comply with the boiling specifications.

It turned out to be a formidable challenge to find a catalyst system, in which only an extremely low loading of a metathesis catalyst (two‐digit ppm level) promotes the ethenolysis of technical‐grade FAMEs with technical‐grade ethylene (3.0, or 99.9 %) at such high efficiency and selectivity. Metathesis catalysts are highly sensitive to the many and strongly varying organic impurities that are present in these substrates.[^47^, ^48^]

In comparative experiments, we studied the activities of various commercially available phoban‐, NHC‐, and CAAC−Ru alkylidenes for the ethenolysis of neat methyl oleate (99 % purity) at 40–60 °C and 10 bar ethylene 3.0. Among them, CAAC−Ru catalysts (Umicore's M1001 and Apeiron's UltraCat (UC)) showed the best results (see Supporting Information, Table S4). Whereas the catalytic performance of both catalysts was similar at a 50 ppm, M1001 showed higher selectivity for the formation of terminal olefins than UltraCat (Table 1 and Table S5 in the Supporting Information). Especially at increased ethylene pressures, M1001 was more active and selective than UltraCat. Under optimal conditions (50 ppm M1001, 40 bar ethylene 3.0, 60 °C, 30 min) 2.5 mmol 99 % methyl oleate gave 91 % conversion and 94 % selectivity, resulting in a turnover number (TON) of >16900. This result goes well beyond the state of the art. For example, Kajetanowicz et al. reported a TON 7624 for a catalyst loading of 50 ppm in the ethenolysis of neat ethyl oleate (90 % purity) with ethylene 3.0.^[48]^

**Table 1:** Selected optimization results for cross‐metathesis of neat methyl oleate (99 %) with ethylene (99.9 %) at 60 °C. “Conv.” stands for conversion, $S_{eth}$ for selectivity towards ethenolysis, TON for turnover number, “UC” for UltraCat. See also Tables S4 and S5 in the Supporting Information.

Catalyst	Load/ppm	p/bar	Time/h	Conv./%	$S_{eth}$ /%	TON
UC	50	10	0.5	79	74	11358
“	”	20	“	87	85	14249
“	”	30	“	87	88	14990
“	”	40	“	87	89	15390
M1001	“	10	”	74	77	11047
“	”	20	“	85	89	14329
“	”	30	“	87	92	15819
“	”	40	“	91	94	16902
“	”	“	1	88	96	16397
“	25	”	0.5	32	98	12401

**Figure 5:**
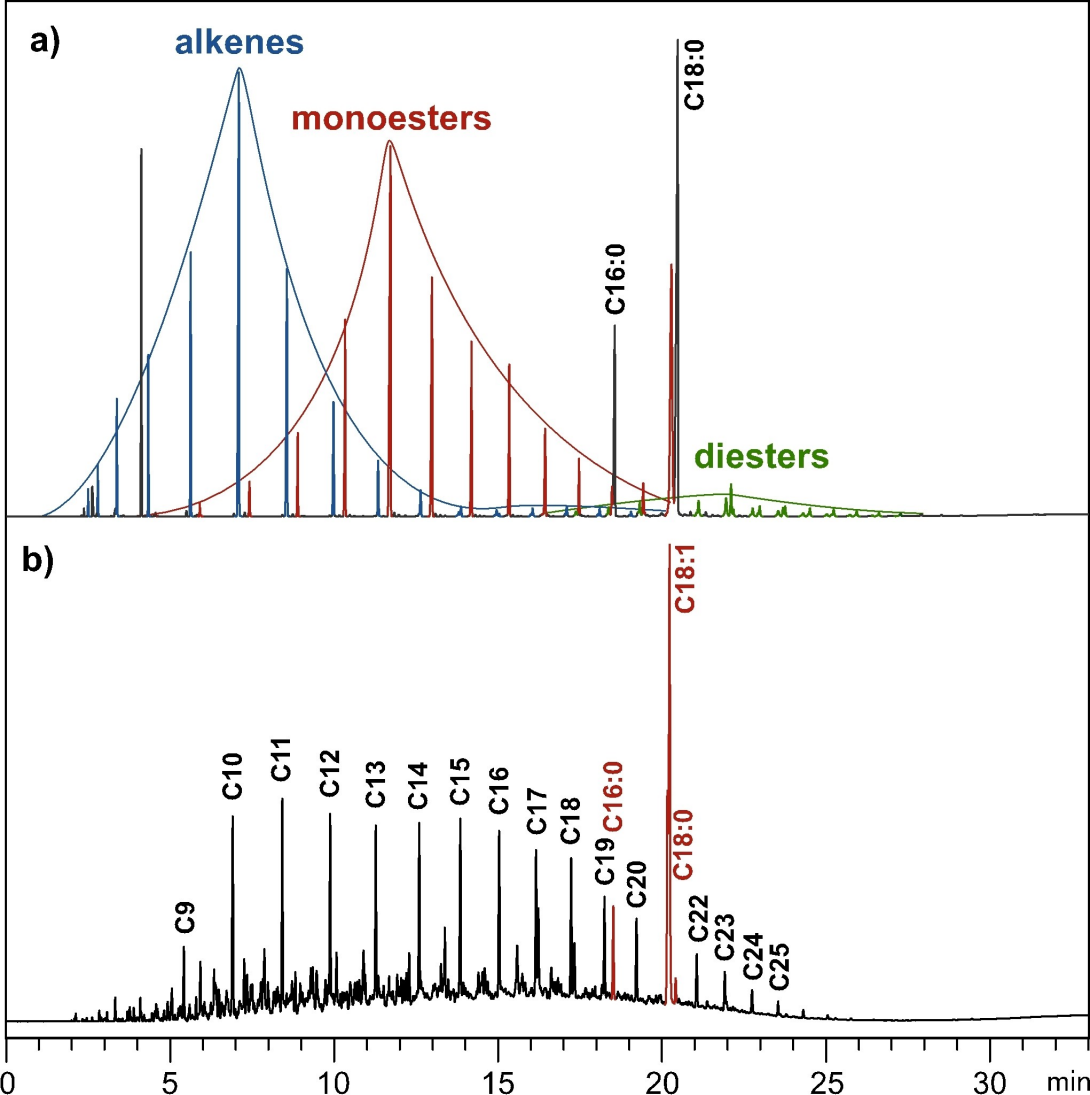
a) Gas chromatogram of the final product mixture (semi‐RME after isomerization and subsequent ethenolysis under optimal conditions); b) gas chromatogram of commercial diesel fuel (peaks highlighted in red denote biodiesel blended into the fuel according to EN 590).

We next conducted a preparative‐scale (250 mmol) ethenolysis of isomerized semi‐RME under the optimized conditions (50 ppm M1001, 40 bar ethylene 3.0, 60 °C). The reaction time was extended to 1 h to make up for the slower heating of the large reaction vessel. Figure 5 shows the stacked gas chromatograms of the product mixture and commercial diesel to visualize the overall similarity, but also some differences. The RME‐product shows a higher proportion of low‐boiling compounds (retention time <10 min), a lower proportion of high‐boiling compounds (retention time 15–20 min), and a higher proportion of C18 : 1 and C18:0 esters. Based on the relative integrals of residual C18 : 1 and methyl palmitate, more than 80 % of the starting material has been converted. Both the 1‐alkenes (blue) and the methyl alkenoates (red) show a bell‐shaped distribution of chain lengths, with the highest signals being 1‐decene and methyl 9‐decenoate. The undesired, high‐boiling diester fraction accounts for less than 5 % of the product mixture. In this respect, the product mixture is different from that obtained via a one‐pot isomerizing ethenolysis of RME. In the latter process, the ethenolysis products undergo further isomerization and metathesis reactions, leading to a broader chain length distribution and a higher content of diesters.^[9]^

The boiling behavior of the product was determined by distilling 100 mL of the product mixture using a distillation analysis apparatus that we constructed according to the EN ISO 3405 (see Supporting Information, Section 6). The boiling point curve obtained this way is shown in Figure 1. It fulfills the requirements of EN 590 that are indicated by the hashed areas, i. e. a maximum 65 % recovery at 250 °C, a minimum 85 % recovery at 350 °C, and a minimum 95 % at 360 °C. As expected from the chromatograms, the boiling point curve is below that of commercial diesel at lower temperatures due to the higher content of low‐boiling components. It rises above that of commercial diesel at >60 % recovery due to its higher content of C18 esters. This effect can be attributed to the high content (19 %) of methyl stearate in the semi‐RME, which cannot undergo ethenolysis. Despite these differences, which can be reduced in the future by optimizing the semi‐hydrogenation, the biodiesel product is still well within the desired specifications.

The cetane index (CI) of the final product was calculated from the boiling point curve following the formula provided in ASTM D976^[11]^ (see Supporting Information, Section 6.2). For our product mixture, a CI of 44 was obtained, which is only slightly lower than the requirement by EN 590 (CI of at least 46). This is because at 50 % recovery, our product mixture boils at 250 °C which is somewhat lower than the boiling temperature of fossil diesel (266 °C). Still, this minor drawback can be eliminated by adding several per cent HVO into our product. At 50 % recovery, HVO boils at 285 °C,^[12]^ therefore its addition will render the cetane index of our biofuel compliant with the EN 590 requirements.

## Conclusions

In summary, a sequence of three reaction steps allows the cost‐efficient transformation of crude RME into a blend of olefins and esters with boiling properties that are compliant with EN 590. In the initial step, poly‐ unsaturated oleic esters are converted into mono‐unsaturated derivatives via Pd‐catalyzed semi‐hydrogenation. This upfront step markedly increases the efficiency of the overall process by avoiding oligomer formation and retarding the catalyst deactivation during the subsequent isomerization. Moreover, it allows to funnel various technical grade oils into this process since biodiesels mostly consist of five C_16_–C_18_ FAMEs, regardless if they are produced from edible RME and soybean oil, waste cooking oil or oils generated from algae and cyanobacteria cultures.^[49]^ We have used edible RME for our experiments, but we would like to emphasize that the use of non‐edible FAME mixtures would be preferrable.

In the next step of our sequence, the double bonds are migrated along the carbon chains by passing the substrate over an inexpensive, Brønsted‐acidic ion exchange resin. Only 100 °C is sufficient for this reaction step. It is crucial to adjust the flow rate in a way that a high proportion of the double bonds migrate more than just one bond from their initial positions, but only a negligible amount of double bonds migrate so far away from the middle that lactone formation occurs. Limiting the average distance of the double bond migration also minimizes the formation of too high‐ and low‐boiling products in the following ethenolysis step, during which the carbon chains are cut at the position of the double bonds by cross‐metathesis with ethylene. We were able to achieve high conversion and a high selectivity for the formation of terminal olefins by using the CAAC‐ruthenium M1001 catalyst at only 50 ppm loading. A high efficiency was reached in this step, so that the amounts of compounds that boil at the beginning (80‐150 °C) and the end of the boiling range (300‐350 °C) could be reduced to a minimum. Starting from crude RME, a product mixture was generated that has a boiling point curve which is well within the boundaries of the EN 590 specifications for petrodiesel.[^9^, ^17^] The low catalyst loadings (20 ppm Pd, 50 ppm Ru), the use of a metal‐free, heterogeneous isomerization catalyst, and the robustness regarding ethylene and fatty ester purity are key advantages of the new process. These results spark optimism that isomerization/ethenolysis sequences can once be used for the industrial production of diesel‐range biofuels. Future steps towards this goal are to conduct semi‐hydrogenation in a continuous‐flow reactor or a CSTR in order to improve the long‐term stability of the heterogeneous isomerization catalyst, as well as to identify long‐lived, heterogeneous catalysts for the ethenolysis of unsaturated esters.

## Supporting Information

Supporting information including all reaction procedures and additional results is available. The data that support the findings of this study are openly available in SciFlection at https://sciflection.com/b1d96a27‐73ff‐46c3‐a91d‐0c84b71fb2b2.

## Conflict of Interests

The authors declare no conflict of interest.

1

## Supporting information

As a service to our authors and readers, this journal provides supporting information supplied by the authors. Such materials are peer reviewed and may be re‐organized for online delivery, but are not copy‐edited or typeset. Technical support issues arising from supporting information (other than missing files) should be addressed to the authors.

Supporting Information

## Data Availability

The data that support the findings of this study are openly available in SciFlection at https://sciflection.com/b1d96a27‐73ff‐46c3‐a91d‐0c84b71fb2b2, reference number 1.
