# Supplementary material for: Sustainable Diesel from Rapeseed Oil Esters by Sequential Semi‐Hydrogenation, Double Bond Isomerization, and Metathesis
Source: Chemistry. 2025 Mar 11;31(22):e202500523. doi: 10.1002/chem.202500523 (PMC12015397; doi:10.1002/chem.202500523)
Supplement: Supplementary file 1 — Supporting Information [file CHEM-31-e202500523-s001.pdf]

Chemistry–A European Journal

Supporting Information

Sustainable Diesel from Rapeseed Oil Esters by Sequential Semi-Hydrogenation, Double Bond Isomerization, and Metathesis

Mykhailo Kondratiuk, Maximilian L. Spiekermann, Thomas Seidensticker, and Lukas J. Gooßen*

Supporting Information

Sustainable diesel from rapeseed oil esters by sequential semi-hydrogenation, double bond isomerization, and metathesis

Mykhailo Kondratiuk,[a] Maximilian L. Spiekermann,[b] Thomas Seidensticker [b] and Lukas J. Gooßen*[a]

[a] *Evonik Chair of Organic Chemistry, Ruhr-Universität Bochum, Universitätsstr. 150, 44801 Bochum, Germany*

[b] *Department for Biochemical and Chemical Engineering, Laboratory for Industrial Chemistry TU Dortmund University, Emil-Figge-Str. 66, 44265 Dortmund, Germany*

***Corresponding author:** lukas.goossen@rub.de

Table of contents

1. Data availability 3
2. General information 3
   2.1. Materials 3
   2.2. Analytic setup 3
   2.3. Calculation of analytic yields by using the relative response factor 3
3. Simulation of the double bond migration distribution 5
4. Synthesis and semi-hydrogenation of FAMEs 7
   4.1. General procedure for RME synthesis 7
   4.2. Semi-hydrogenation 7
5. Double bond isomerization 9
   5.1. Flow reactor construction 9
   5.2. Reaction procedure 9
   5.3. Analysis procedures 11
   5.4. FAME isomerization results 11
6. Ethenolysis 14
   6.1. Substrate pre-treatment 14
   6.2. Model substrate ethenolysis optimization 14
      6.2.1. Reaction procedure 14
      6.2.2. Optimization results 16
   6.3. Large-scale ethenolysis 19
7. Distillation analysis 21
   7.1. Experimental distillation analysis 21
   7.2. Cetane index 25
   7.3. Simulated distillation analysis 25
8. Attempts of subsequent hydrotreatment towards kerosene 28
   8.1. Aviation kerosene specifications 28
   8.2. Experimental details 28
   8.3. Experimental results 30
9. References 32

1. Data availability

All experiments, together with analytic data and calculation spreadsheets can be found under: https://sciflection.com/b1d96a27-73ff-46c3-a91d-0c84b71fb2b2.

Alternatively, the data can be found at sciflection.com under the DOI of this publication.

2. General information

2.1. Materials

All materials, unless otherwise stated, were purchased at general chemical suppliers and used as received. Ru alkylidene catalysts were donated by Umicore AG & Co. KG, HOSOME used for semi-hydrogenation was donated by DAKO AG. RME was obtained upon transesterification of canola oil. Ethylene was purchased at Air Liquide and has a purity of 99.9% (Ethylene 3.0).

2.2. Analytic setup

Gas chromatograms were recorded at Hewlett-Packard 6890 GC series gas chromatographs with FID detectors, one equipped with an HP-5 capillary column (30 m×0.250 mm×0.25 µm) and another with a J&W DB-FastFAME capillary column (60 m×0.250 mm×0.25 µm). Mass spectra were recorded using Agilent 8890 GC series gas chromatograph equipped with an HP-5-MS UI (30 m×0.250 mm×0.25 µm) capillary column and Agilent 5977 GC/MSD mass detector. Signal analysis and quantification was done using default Agilent GC solutions and GCMS solutions software. Simulated distillation analysis was done using Envantage Dragon II SimDis software (30-day test version) with the D2887 method (for detailed instructions, see below).

For HP-5 & HP-5MS columns, the following temperature program was used: Carrier gar N_2, split 35:1 (10:1 for ozonolysis samples), constant flow 1.2 mL/min, injector temperature 220 °C, detector temperature 300 °C. Oven temperature was held at 40 °C for 6 min, then raised to 250 °C at a rate 15.0 °C/min and then held for 7 min. Integration was done using advanced baseline correction, peak/valley ratio 500, slope sensitivity 50, peak with 0.1. For hydrogenated samples, slope sensitivity 100 and peak width 0.05 were used.

For DB-FastFAME column, following temperature program was used: Carrier gar N_2, split 5.0:1, constant pressure 20 psi, injector temperature 280 °C. Oven temperature was held at 150 °C for 5 min, then raised to 210 °C at a rate 1.0 °C/min and then held for 3 min. Integration was done using advanced baseline correction, peak/valley ratio 500, slope sensitivity 1, peak width 0.1.

2.3. Calculation of analytic yields by using the relative response factor

The response factor of a substance in chromatographic analysis is defined as follows:

$$F = \frac{Area}{m * \eta} \tag{1}$$

m: analyte weight; η: analyte purity

To analyze the ethenolysis reaction mixtures, relative response factors (RRF) of all reaction components against the internal standard had to be determined:

$$RRF = \frac{F_1}{F_{Std}} = \frac{Area_1 * m_{Std} * \eta_{Std}}{Area_{Std} * m_1 * \eta_1} \tag{2}$$

M_1: molar mass of analyte 1 ($g*mol^{-1}$); n_1: quantity of 1 (mol).

Quantity of the analyte based on a known amount of the internal standard was thus calculated using the following formula:

$$n_1 = \frac{Area_1 * m_{Std} * \eta_{Std}}{Area_{Std} * \eta_1 * M_1 * RRF} \tag{3}$$

For the isomerization and the hydrotreatment experiments, due to small structural differences between the educts and the products and due to a low availability of all precursors at high purities, the RRF values were calculated. For the ethenolysis experiments, the RRF values were determined experimentally.

Procedure for experimentally determining relative response factors

Five 1.5 mL GC vials were charged with the standard (standards) and the analytes (Table S1) and subsequently filled up with EtOAc.

Table S1. Amounts of the analytes and the internal standard(s) to be filled in the vials.

	Amount each analyte	*Amount standard(s)*
1)	~20 mg	~40 mg
2)	~40 mg	~40 mg
3)	~60 mg	~40 mg
4)	~80 mg	~40 mg
5)	~100 mg	~40 mg

An aliquot (0.125 mL) from each vial was diluted with 3 mL EtOAc; from the new solutions, 0.500 mL were transferred into another GC vial which was filled up with EtOAc. These vials were submitted to a GC analysis using the analysis procedure that was to be used for the separation of the targeted reaction mixture. The RRF values were determined using the formula (1).

Calculation of theoretical relative response factors

The theoretical response factors were calculated as a fraction of the carbon content of each analyte and the carbon content of the internal standard.

The carbon content (CC) was determined as follows:

$$CC_1 = \frac{N(C)_1 * M(C)}{M_1} \tag{4}$$

Where $N(C)_1$ is the number of carbon atoms in the analyte 1, $M(C)$ is the atomic mass of carbon, and M_1 is the molecular mass of the analyte 1.

3. Simulation of the double bond migration distribution

The simulation was written in Python and can be visualized in such applications as e.g. MatPlotLib on Binder.

```
import numpy as np
import matplotlib.pyplot as plt

# Define constants
num_molecules = 100000  # Number of molecules to simulate
alpha = 0.0896  # Parameter for Maxwell-Boltzmann
start_position = 9  # Initial position of the double bond
min_position = 2  # Lower boundary
max_position = 17  # Upper boundary

# Generate number of steps using Maxwell-Boltzmann distribution
def maxwell_boltzmann(v):
    return v**2 * np.exp(-alpha * v**2)

# Create discrete step numbers (0 to 15) following the Maxwell-Boltzmann probability
step_numbers = np.arange(0, 16)  # Include 0 steps as well
probabilities = maxwell_boltzmann(step_numbers)
probabilities /= np.sum(probabilities)  # Normalize

# Randomly assign step counts to each molecule
random_values = np.random.random(num_molecules)
quantized_steps = np.digitize(random_values, np.cumsum(probabilities)) - 1  # Shift to match 0 index

# Simulate movement of the double bond
final_positions = np.full(num_molecules, start_position)

for i in range(num_molecules):
    position = start_position
    steps = quantized_steps[i]

    for _ in range(steps):
        if np.random.rand() < 0.5:
            position -= 1  # Move left
        else:
            position += 1  # Move right

        # Reflect at boundaries
        if position < min_position:
            position = min_position + 1
        elif position > max_position:
            position = max_position - 1

    final_positions[i] = position

# Compute histograms
hist_values, bins = np.histogram(final_positions, bins=np.arange(1, 19), density=True)
step_hist_values, step_bins = np.histogram(quantized_steps, bins=np.arange(0, 17), density=True)

# Compute lengths of olefin and ester after ethenolysis
olefin_lengths = final_positions + 1
ester_lengths = (max_position + 1) - final_positions

# Histograms for olefin and ester lengths
olefin_hist, olefin_bins = np.histogram(olefin_lengths, bins=np.arange(2, 20), density=True)
ester_hist, ester_bins = np.histogram(ester_lengths, bins=np.arange(2, 20), density=True)
```

```
# Actual boiling points
olefin_boiling_points = np.array([-103.8, -47.7, -6.2, 30, 63, 94, 121, 147, 172, 193, 213, 235, 252, 270,
287, 302, 317], dtype=float)
ester_boiling_points = np.array([np.nan, 78.6, 101.9, 127.36, 151, 169.7, 194.1, 212.3, 233, 248, 268,
288, 295, 308.7, 324, 337, 353], dtype=float)

valid_olefin_indices = np.arange(2, 19)
valid_ester_indices = np.arange(2, 19)

# Print the distribution for steps (in percentage)
print("Average number of steps")
average_value = np.sum(step_hist_values * (step_bins[:-1] + step_bins[1:]) / 2)
print(average_value)
print(" ")


# Print probability of number of steps
print("\nProbability of number of steps:")
for i in range(len(step_hist_values)):
    print(f"{i} steps: {step_hist_values[i] * 100:.2f}%")

# Print distribution of double bond positions
print("\nDistribution of double bond position:")
for i in range(len(hist_values)):
    print(f"Position {i + 1}: {hist_values[i] * 100:.2f}%")

# Print distribution of olefin lengths
print("\nLength Olefin after ethenolysis:")
for i in range(len(olefin_hist)):
    print(f"{olefin_bins[i]:.0f}: {olefin_hist[i] * 100:.2f}%")

# Print distribution of ester lengths
print("\nLength Ester after ethenolysis:")
for i in range(len(ester_hist)):
    print(f"{ester_bins[i]:.0f}: {ester_hist[i] * 100:.2f}%")

# Generate Maxwell-Boltzmann values for overlay (continuous function)
v_values = np.linspace(0, 15, 500)  # Range of v from 0 to 15
boltzmann_y = maxwell_boltzmann(v_values)  # Continuous Maxwell-Boltzmann function
boltzmann_y /= np.max(boltzmann_y)  # Normalize to the same scale as histograms

# Print the distribution for Maxwell-Boltzmann function
print("Maxwell-Boltzmann (in %):")
boltzmann_results = "\n".join(
    [f"v = {v:.2f} P(v) = {p*100:.2f}" for v, p in zip(v_values, boltzmann_y* max(hist_values))]
)
print(boltzmann_results)
print(" ")

# First plot: Double bond position distribution
plt.figure(figsize=(10, 6))
plt.bar(np.arange(1, 18) + 0.5, hist_values, color='blue', alpha=0.6, label='Double Bond Positions',
width=0.8)
plt.plot(v_values, boltzmann_y * max(hist_values), color='red', linewidth=2, label='Maxwell-Boltzmann
(Line)')
plt.bar(step_bins[:-1] + 0.5, step_hist_values, width=0.8, color='green', alpha=0.6, label='Step
Distribution')
plt.xlabel('Final Double Bond Position')
plt.ylabel('Probability Density')
plt.title('Double Bond Position Distribution vs. Maxwell-Boltzmann')
plt.xticks(np.arange(1, 18))
plt.legend()
```

```
plt.grid(axis='y', linestyle='--', alpha=0.7)
plt.show()

# Second plot: Overlay of Olefin and Ester Length Distributions
plt.figure(figsize=(10, 6))

# Plot olefin length distribution (red bars)
plt.bar(olefin_boiling_points[valid_olefin_indices - 2], olefin_hist, width=10, color='red', alpha=0.6, label='Olefin Length')

# Plot ester length distribution (blue bars)
ester_boiling_values = ester_boiling_points[valid_ester_indices - 2]
valid_ester_mask = ~np.isnan(ester_boiling_values)

plt.bar(ester_boiling_values[valid_ester_mask] + 3.5, ester_hist[valid_ester_mask], width=10, color='blue', alpha=0.6, label='Ester Length')

plt.xlim(50, 350)
plt.xlabel('Boiling points / °C')
plt.ylabel('Relative content')
plt.title('Simulated distribution of ethenolysis products ')
plt.legend()
plt.grid(axis='y', linestyle='--', alpha=0.7)
plt.show()
```

4. Synthesis and semi-hydrogenation of FAMEs

4.1. General procedure for RME synthesis

A 1 L round-bottomed flask equipped with a Teflon-coated stir bar was charged with methanol (146 mL, 3.6 mol, 12.0 equiv.) and NaOH (0.6 g, 1.5 mmol, 0.05 equiv.). The mixture was stirred until full dissolution of the base. Then, rapeseed oil (291 mL, 0.3 mol, 1.00 equiv.) was added and the mixture was vigorously stirred at 60 °C for 3 h. Subsequently, the reaction mixture was poured into a 1 L separating funnel. After it separated in two phases, the bottom phase was discarded. The top phase was washed with water (2 × 300 mL + 50 mL 1M aq. HCl each time) and brine (1 × 200 mL), dried over $MgSO_4$ and decanted. Distillation under reduced pressure (5 mbar, head-T 180-190 °C) afforded RME as a clear, slightly yellowish liquid.

4.2. Semi-hydrogenation

Semi-hydrogenation of FAMEs was performed according to Behr et al.[1] It is catalyzed by solvent-stabilized Pd nanoparticles, which are formed via an autoreduction of $Pd(OAc)_2$ or $PdCl_2$ in propylene carbonate.

Nanoparticle solution preparation: $Pd(OAc)_2$ (11.2 mg, 0.05 mmol) was dissolved in propylene carbonate (51 g, 42.2 mL, 0.5 mol) to afford a 0.01 mol-% Pd solution. The solution was stirred at 80 °C for 2 h at 800 RPM which caused an autoreduction of the palladium with the formation of a grey, homogeneous solution of nanoparticles. With constant stirring, the mixture was cooled down to 15 °C. Analysis by transmission electron microscopy confirmed that the nanoparticles formed this way have a narrow diameter distribution around 4 nm.

Semi-hydrogenation procedure: An overhead-stirred 300 ml glass reactor was charged with 5 g of the Pd-nanoparticle solution (0.005 mmol Pd, 20 ppm vs. molar amount of the FAME) and 20 g of propylene carbonate, pressurized with 10 bar and heated to 80 °C to pre-activate the catalyst. Then, the FAME (85,2 mL, 0.25 mol) was added, and the reaction mixture was stirred at 1800 rpm for 2 h while

maintaining a constant hydrogen pressure of 10 bar and a temperature of 80 °C. Then, the reaction was cooled to 15°C, excess hydrogen was slowly released, and the stirrer was stopped to allow the layers to separate. The upper layer, which contained the semi-RME, was separated off, analyzed using GC-FID (DB FastFAME column) and purified via distillation under reduced pressure (5 mbar, head-T 180-190 °C).

Due to the low solubility of the Pd nanoparticles in FAMEs compared to propylene carbonate, at best traces of the used 20 ppm Pd leak into the product phase. Its Pd-content was below the detection limit of our instruments (ICP-OES).

Results

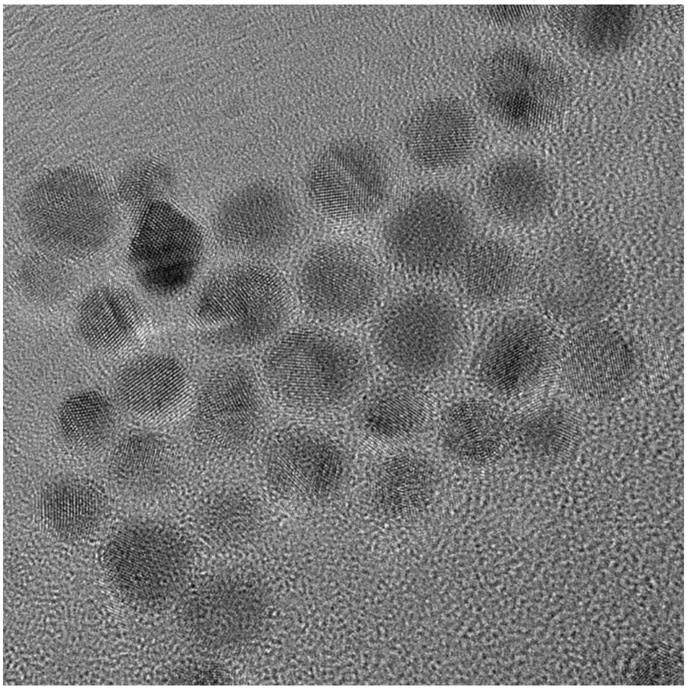

Figure S1. TEM-image of the Pd nanoparticles obtained upon the method described above.

The compositions of the semi-hydrogenated FAME substrates and their non-hydrogenated educts are presented in the table below:

Table S2. Composition of the FAME substrates used in this work, as determined via GC-FID using the DB FastFAME column. The positions of the C18:1 double bonds were determined using ozonolysis-reduction (see Section 5.3 and KOM-1735).

Substrate	HOSOME	semi-HOSOME	RME	semi-RME
C16:0 / %	3.5	3.4	4.6	4.8
C18:0 / %	3.1	5.6	1.7	18.5
C18:1 *cis/trans-8* / %	-	3.4	-	10.1
C18:1 *cis/trans-9* / %	87.1 (*cis*)	81.9	65.1 (*cis*)	42.0
C18:1 *cis/trans-10* / %	-	3.6	-	11.4
C18:1 *cis/trans-11* / %	-	1.4	-	6.7
C18:1 *cis/trans-12* / %	-	0.8	-	5.9
C18:2 / %	6.3	-	19.8	-
C18:3 / %	-	-	8.8	-
Total UFA / %	93.4	91	93.7	76.6

5. Double bond isomerization

5.1. Flow reactor construction

For the continuous flow experiments, we used a self-made flow reactor that was successfully used in our previous work.[2,3] The reactor consists of a 280 mm stainless steel tube (V4A or 316 stainless steel) with 0.50-inch outer, and 10.0 mm inner diameter embedded in a cylindrical heating mantle (220 mm × 40 mm) milled from aluminium. The heating mantle has two slots for heating cartridges coaxial to the reactor tube, and multiple slots for temperature measurement (Figure S2). The heating mantle together with the reactor is surrounded by a stone wool insulation mantle (280 mm × 40 mm inner diameter). The heating cartridges are controlled with a commercially available digital temperature controller equipped with a K-type thermocouple. The self-learning heating algorithm of the controller in combination with the insulated heat conductive aluminium mantle ensures homogeneous heat distribution along the catalyst bed with deviations below 5 °C over the entire length of the reactor.

During operation, the reactor was positioned vertically, and the substrate feed was delivered by a Kontron HPLC pump (Figure S3).

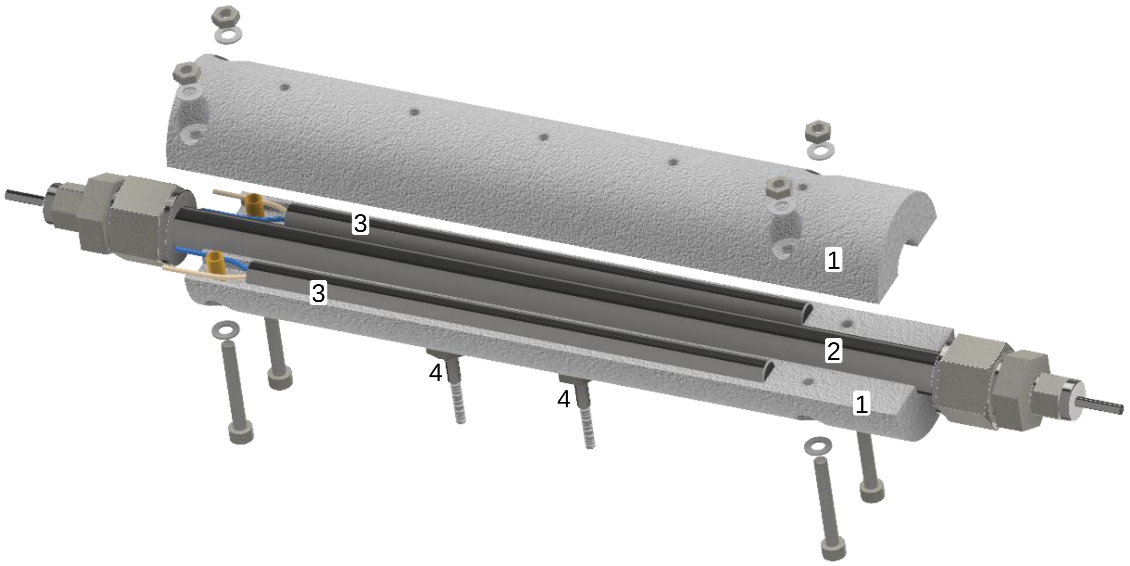

Figure S2. Schematic drawing of the flow reactor, designed in Autodesk Inventor. 1) Aluminium heating mantle; 2) reactor tube with Swagelok® connectors; 3) heating cartridges; 4) the main and the safety thermocouples.

5.2. Reaction procedure

All FAME feedstock was distilled at 5 mbar prior to isomerization to remove water, colourants, and eventually present glycerin or other solvents. Ion-exchange resins, which are commercially available mostly in their wet form, were dried overnight at 10^{-3} mbar and 120 °C. Amberlyst 15 dry was also dried at 10^{-3} mbar for at least 2 h to remove residual moisture.

The reactor tube was filled with a glass wool plug of about 2 cm in length; then, 10.0 g ion-exchange resin was filled in. The residual inner space was filled up with a second glass wool plug. The reactor tube was closed, weighed, then filled with the substrate and weighed again. The weight difference was used to calculate the free reactor volume and thus the mean residence time.

The reactor tube was positioned inside the heating mantle, connected to the substrate feed, and equilibrated at the set temperature and the set flowrate for three mean residence times. Then, the product was collected into weighed headspace vials. During the experiment, every 30 min (for flow

rates 0.1–0.3 mL) or every 15 min (for higher flow rates) the receiving vial was changed, weights of the starting material reservoir and full receiving vial were recorded, and 2x two-droplet samples were taken from the reactor exit tube. One sample was hydrogenated, the other one was ozonolysed.

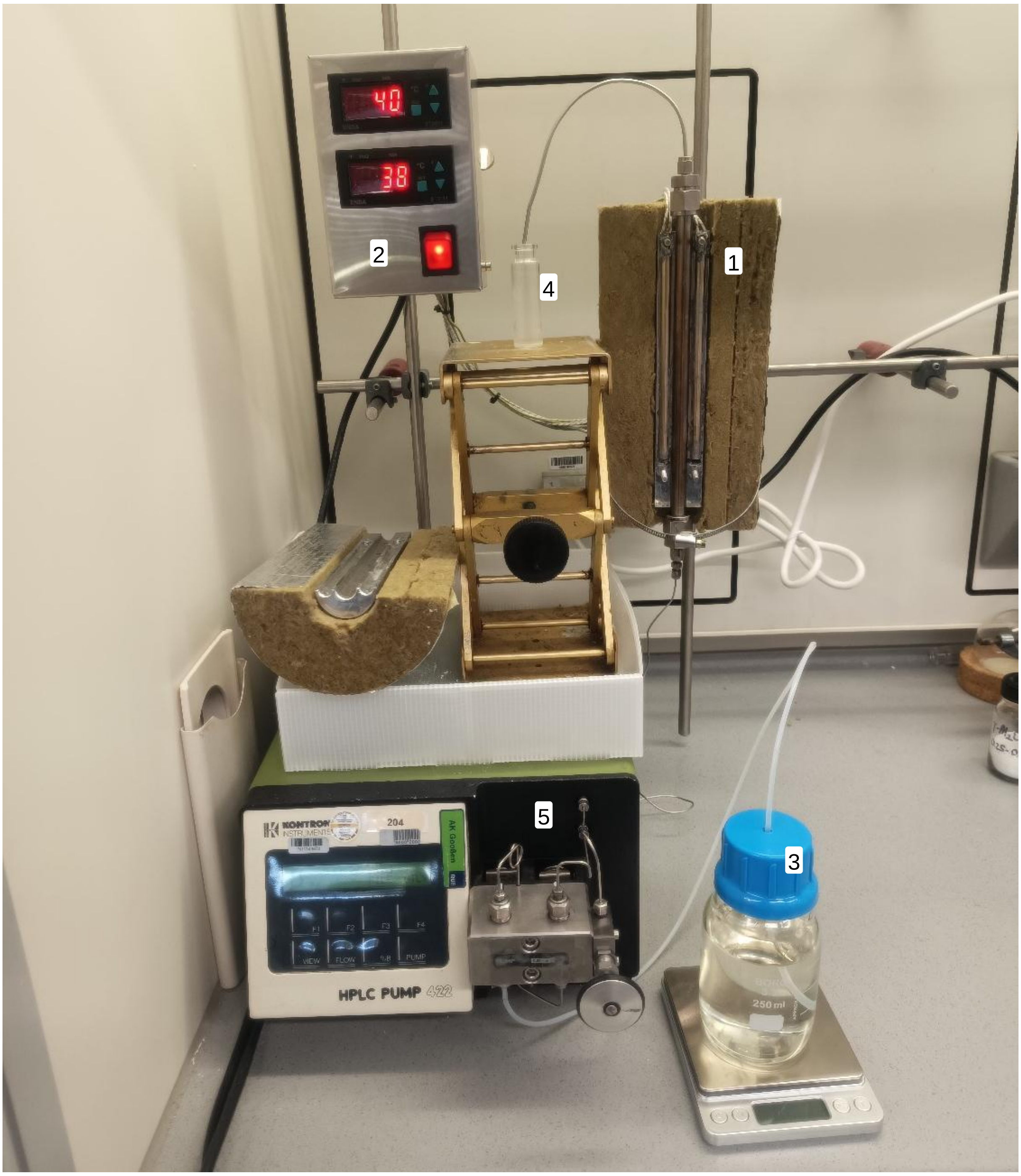

Figure S3. The flow reactor in operation. 1) The reactor in a stone wool insulation mantle (front half removed for demonstration purposes); 2) temperature controller, with the main controlling element and the safety controller; 3) substrate container on the balance; 4) substrate collection vial; 5) HPLC pump

The analysis workflow was as follows:

1) The starting material was analyzed on a DB FastFAME column to determine exact composition of the substrate. A sample of the starting material was hydrogenated to determine the ratio between the resulting methyl stearate and methyl palmitate which is initially contained in the substrate. Initially contained methyl stearate was mathematically subtracted based on FastFAME analysis values.
2) The yields of linear and branched C18:1 isomers, as well as yields of side products were hydrogenating the product mixture's two-droplet aliquot and recording gas chromatograms using an HP-5 column. Integration of the signals versus methyl palmitate, followed by comparison of these values to the starting material, gave the value of the overall monomer content.
3) Another two-droplet aliquot of the product mixture were subjected to ozonolysis, followed by reductive workup (Zn/HOAc). The GC integrals (HP-5 column) of corresponding aldehydes were used to determine the distribution of double-bond isomers among the total amount of linear isomers.
4) Weights of the receiving vials were compared with weight change of the substrate container to determine mass balance and effective flow rate

In general, the analysis algorithm is almost identical to the procedure described in our previous publication,[2] but here we omit distillation of the product fractions since we have proven that the results based on hydrogenated sample analysis correlate with the results obtained upon distillation. This allowed us to move towards momentary droplet-wise aliquots instead of analysis of a sample that is collected over an extended period.

5.3. Analysis procedures

Hydrogenation procedure. A 2 mL GC vial containing a two-droplet reaction mixture sample was charged with a small stir bar, 0.5 mL EtOAc, and a spatula tip of palladium on coal (10% Pd). The vial was placed in an autoclave, the latter was sealed and pressurized with H_2 (20 bar). The reaction mixture was stirred at 80°C for 30 min. Subsequently, the entire reaction mixture was transferred in a vial containing 3 mL EtOAc and 2 mL 0.5 mol-% aq. $NaHCO_3$. The two-phase mixture was vigorously mixed with a Pasteur pipette, and 0.25 mL organic fraction were pushed through a short silica/$MgSO_4$ plug into a GC vial, which was then filled up with EtOAc. The resulting sample was analyzed via GC-FID.

Ozonolysis procedure. A 2 mL GC vial containing a two-droplet reaction mixture sample was charged with a small stir bar and 0.5 mL MeOH, cooled down at -18 °C (ice/salt bath), and ozonolysed with an oxygen-ozone stream (22-24 mL/min, ca. 5.6 g O_3 per L O_2) at -18 °C for 15 min. Then, the vial was taken out of the cold bath and charged with 0.05 g Zn and glacial 0.50 mL acetic acid and stirred vigorously at r.t. for another 5 min. Subsequently, the entire reaction mixture was transferred in a vial containing 3 mL EtOAc and 2 mL 0.5 mol-% aq. $NaHCO_3$. The two-phase mixture was vigorously mixed with a Pasteur pipette, and 0.50 mL organic fraction were pushed through a short silica/$MgSO_4$ plug into a GC vial, which was then filled up with EtOAc. The resulting sample was analyzed via GC-FID.

5.4. FAME isomerization results

The space-time-yield for the optimal conditions was calculated as the flow of the product (quantitative yield) in kg per hour, divided by the volume of the catalyst bed. The catalyst bed of Amberlyst 15 took up ca. 25 cm of the reactor length.

Below, additional double bond isomerization results are included. The catalyst screening (Figure S4) was initially done with HOSOME, until semi-HOSOME became available. Then, the two best results were reproduced with semi-HOSOME. Notably, the Amberlyst-type catalysts resulted in formation of 7-8% oligomers which is the amount of linoleic acid in HOSOME. Upon switching to the semi-hydrogenated substrate, the amount of oligomers was diminished to traces with Amberlyst 36. With Amberlyst 15, the overall image of the product distribution (e.g. 9% γ-stearolactone) clearly speaks of a higher activity with semi-HOSOME. This clearly shows that multiply unsaturated FAMEs show negative impact onto the catalytic activity of divinylbenzene-crosslinked resins. This impact is probably resulted by reaction between FAMEs and divinylbenzene under acidic conditions, which leads to irreversible passivation of the catalyst's surface.[4]

The condition optimization and substrate screening results, as well as the long-term experiment results for semi-RME are shown in the main publication manuscript. Figure S5 shows the long-term experiment results for semi-HOSOME. Similar to data in Figure S4, presence of multiply unsaturated FAMEs leads to a rapid decay in catalytic performance of Amberlyst 15.

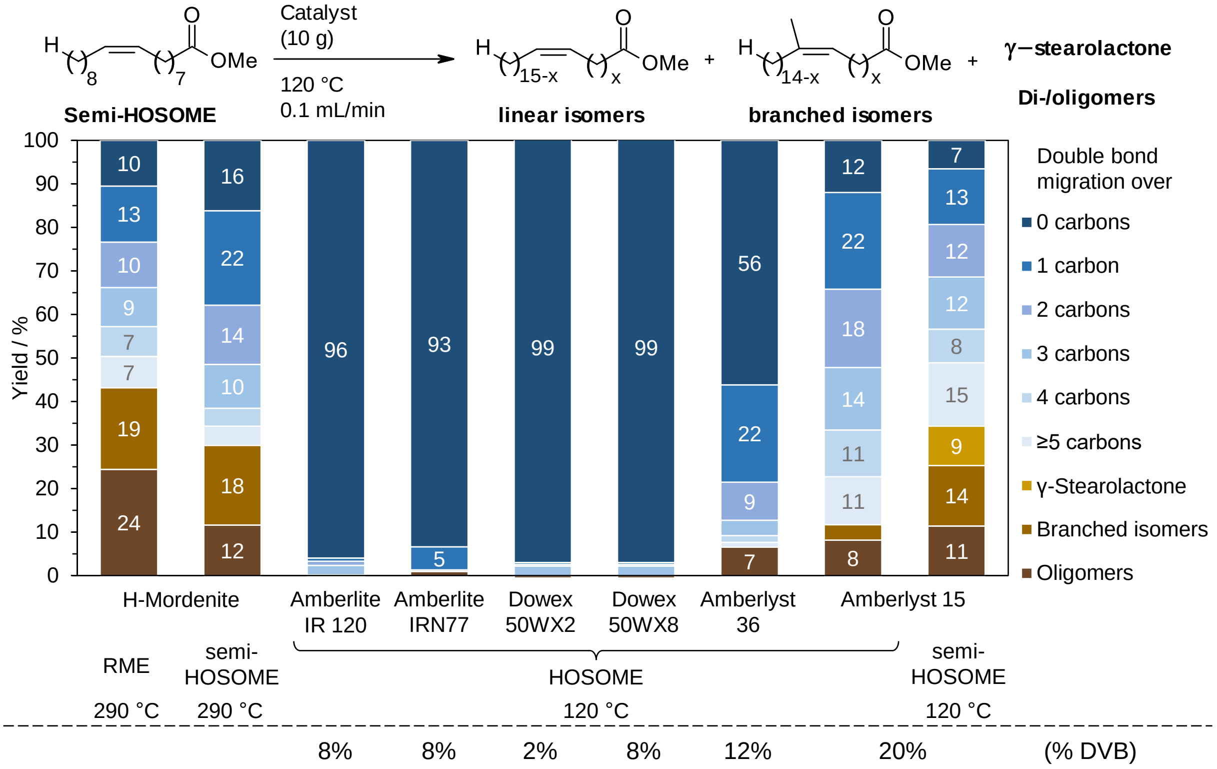

Figure S4. Results of a catalyst screening (H-Mordenite vs. various ion-exchange resins) as well as brief substrate screening (RME vs. semi-HOSOME). Stacked column blocks with yields below 5% are not labelled due to space restrictions. Flowrate 0.1 mL/min corresponds to a mean retention time of 150 min and a space-time of 0.27 $kg \cdot L^{-1} \cdot h^{-1}$. For the ion-exchange resin results, the percentage of divinylbenzene (cross-linker) specified underneath each catalyst, according to the data sheets provided by manufacturer.

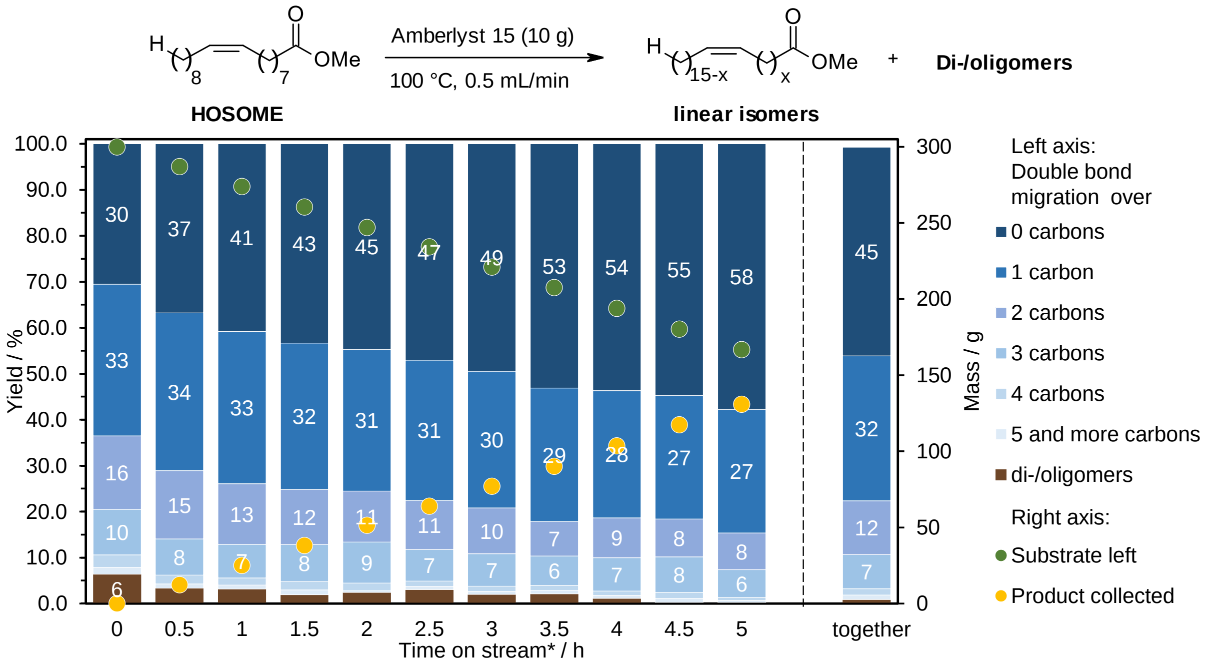

Figure S5. Results of a long-term isomerization experiment. Conditions: HOSOME, 10 g Amberlyst 15 dry, 100 °C, 0.5 mL/min. Resulting productivity: 1.33 kg·L^{-1}·h^{-1}. *After equilibration for 3 mean residence times.

Table S3. Assignments of the double-bond isomerization results to the corresponding experiments in the repository. The detailed reaction procedures, as well as calculation spreadsheets are contained within each repository file.

Catalyst	Substrate	Temperature / °C	Flowrate / mL·min^{-1}	Experiment - #
Amberlite IR 120	HOSOME	120	0.1	KOM-1090
Amberlite IRN77	"	"	"	KOM-1092
Dowex 50W X2	"	"	"	KOM-1093
Dowex 50W X8	"	"	"	KOM-1100
Amberlyst 36	"	"	"	KOM-1085
Amberlyst 15	"	"	"	KOM-1089
Amberlyst 36	semi-HOSOME	"	"	KOM-1434
Amberlyst 15	"	"	"	KOM-1565
"	"	100	"	KOM-1567
"	"	"	0.2	KOM-1570
"	"	"	0.3	KOM-1573
"	"	"	0.4	KOM-1574
"	"	"	0.5	KOM-1578
"	"	"	0.6	KOM-1595, 1624
"	"	"	0.7	KOM-1652
"	semi-RME	"	0.5 (long-term)	KOM-1654
"	HOSOME	"	0.5 (long-term)	KOM-1564
"	RME	"	0.5	KOM-1671

6. Ethenolysis

6.1. Substrate pre-treatment

Preparation of methyl oleate (MO, 99% purity) and methyl elaidate (ME, 97%purity) for the model reaction screening: the substrate was mixed with 20 wt.-% activated neutral alumina in a flame-dried glass vial, crimped, and degassed by evacuation at 10^{-3} mbar for 2 h. Subsequently, the vial was filled with nitrogen and stored overnight.

Collected fractions of isomerized semi-RME were combined, distilled, and treated the same way as methyl oleate.

Toluene and *trans*-decalin were dried and degassed by mixing with 3Å activated molecular sieves (10 wt.-%) and sparging with nitrogen for 30 min.

The resulting water content of dried liquids was measured on a Karl Fischer titrator and was found to be below 5 ppm for FAME samples and below 3 ppm for toluene and decalin.

All pre-treated substrates were stored in a nitrogen-filled glovebox (<0.5 ppm O_2, <0.5 ppm H_2O).

6.2. Model substrate ethenolysis optimization

6.2.1. Reaction procedure

Stock solutions: a 10 mL oven-dried headspace vial was charged with the catalyst (50x scale) and transferred into a nitrogen-filled glovebox. In the glovebox, it was charged with 5.0 mL toluene. For the 50x MO:standard stock solution, a 100 mL oven-dried headspace vial was charged in the glovebox with 42.8 mL methyl oleate (99% purity, 125 mmol, 1.0 equiv.) and 5.0 mL *trans*-decalin (30.9 mmol, 0.247 equiv.).

Reaction procedure: in the glovebox, an oven-dried 10 mL headspace vial equipped with an oven-dried, Teflon-coated stir bar was charged with 0.96 mL MO:standard stock mixture (2.5 mmol scale reaction), and 100 µL catalyst stock solution (for a 50 ppm catalyst load). Then, the vial was quickly crimped, equipped with a helically woven syringe needle, and placed in a steel autoclave. The autoclave was sealed, taken out of the glovebox, pressurized with ethylene 3.0 and placed in a preheated heating block (**Figure S6**). It was then stirred at the set temperature for the set time. Subsequently, the autoclave was opened, the mixture was diluted with 5 mL EtOAc, and 0.25 mL were transferred into another vial charged with 3 mL EtOAc and 2 mL 0.5 mol-% aq. $NaHCO_3$. The two-phase mixture was vigorously mixed with a Pasteur pipette, and 0.25 mL organic fraction were pushed through a short silica/$MgSO_4$ plug into a GC vial, which was then filled up with EtOAc. The resulting sample was analyzed via GC-FID.

During the reaction optimization, reactions that required the same temperature and pressure setting were conducted in the same autoclave simultaneously to minimize possible errors.

Ethenolysis of isomerized FAME samples

The preparation of the stock solution and the synthetic procedure was identical to the one described above. The “purity” of the isomerized FAMEs was calculated on a basis of C18:1 isomers content in the sample after the isomerization reaction.

Upon the reaction end, 0.5 mL of the reaction mixture was transferred into a GC vial and diluted with 1.0 mL CS_2. Then, a gas chromatogram for a simulated distillation analysis was recorded (temperature program – see below).

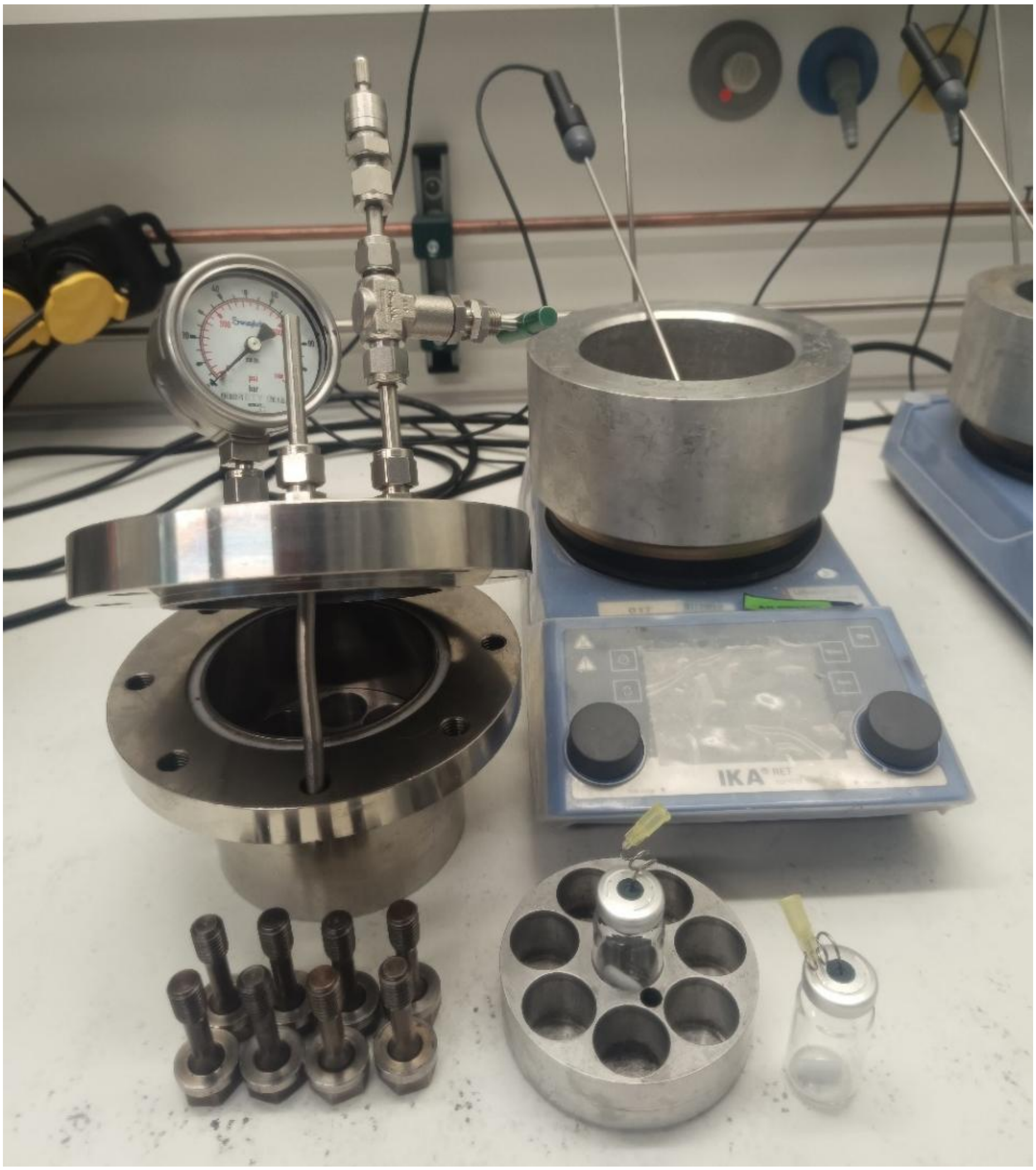

Figure S6. The V4A steel (also known as 316 steel) autoclave used for the ethenolysis reaction, that can fit an aluminium vial block with 8 vial slots. Top right: IKA magnetic stirring hotplate, with an aluminium heating block designed to snuggly fir the autoclave to the left. Bottom right: 10 mL headspace vials equipped with a Teflon-coated stir bar, an aluminium cap fitted with a Teflon-coated butyl rubber septum, and a helically bent syringe needle.

6.2.2. Optimization results

To find the optimal conditions, we first performed a reaction optimization with 99% methyl oleate (MO) (Scheme S1). We found that for a neat reaction, a catalyst load of 50 ppm is an optimal value that would enable decent comparison between different catalyst types and allow optimization of total conversion up to 91%. For the initial catalyst screening, we used the library of Ru alkylidene catalysts. Besides standard **G2** and **HG2** catalysts, this library contained a Phoban-ligated **M110** and three CAAC-ligated catalysts **M1001**, **M1002**, and **UltraCat**.

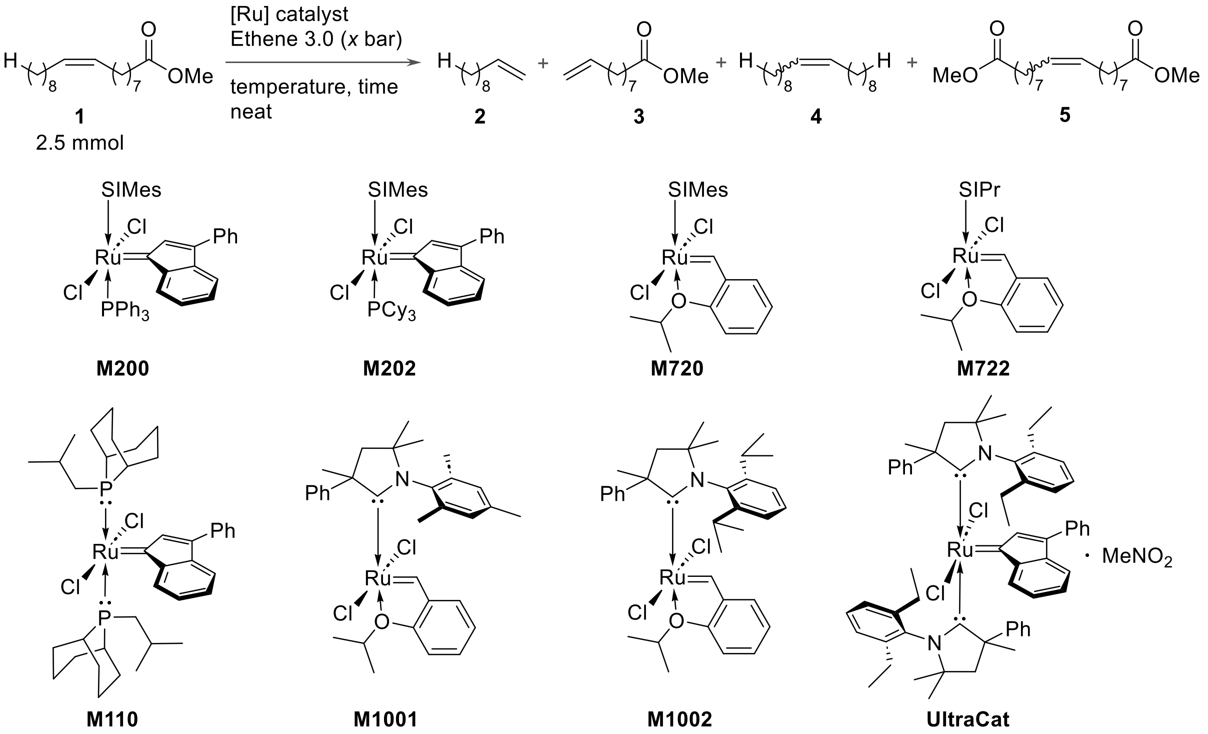

Scheme S1. Methyl oleate ethenolysis reaction overview, with all possible products and the catalysts that were used in this work.

Table S4. Catalyst screening results for methyl oleate ethenolysis. **2** stands for 1-decene, **3** for methyl dec-9-enoate, "conv." for conversion, S_{eth} for selectivity towards ethenolysis, "mass bal." for mass balance. Substrate: methyl oleate. Internal standard: *trans*-decalin.

Exp. - #	Catalyst	Loading / ppm	T / °C	p / bar	Time / h	**2** / %	**3** / %	Conv. / %	S_{eth} / %	TON	Mass bal. / %
1579	**M110**	50	40	10	0.5	3	3	5	100	502	97
1580	**M200**	"	"	"	"	7	7	52	14	1365	97
1581	**M202**	"	"	"	"	0	0	1	n.d.	n.d.	99
1582	**M720**	"	"	"	"	8	8	55	16	1669	96
1583	**M722**	"	"	"	"	24	24	60	42	4793	97
1584	**M1001**	"	"	"	"	50	52	65	80	10205	98
1585	**M1002**	"	"	"	"	1	1	3	100	269	98
1586	**UltraCat**	"	"	"	"	1	1	3	100	161	97
1596	**M110**	"	60	"	"	15	15	18	100	3044	97
1597	**M1001**	"	"	"	"	55	56	74	77	11047	98
1598	**M1002**	"	"	"	"	52	53	59	94	10501	97
1599	**UltraCat**	"	"	"	"	56	57	79	74	11358	98

As it can be seen in Table S4, the CAAC-ligated Ru alkylidenes **M1001**, **M1002**, and **UltraCat** afford comparable ethenolysis yields at 60 °C and 10 bar. Since the yield differences were marginal, we decided to find the optimal conditions for each catalyst separately. The optimization results are shown in Table S5; the highest TON at 50 ppm catalyst loading were achieved for **M1001** which afforded 91% conversion and 94% ethenolysis selectivity at 60 °C and 40 bar ethylene.

Table S5. Ethenolysis optimization results for **M1001, M1002,** and **UltraCat**. "Conv." stands for conversion, S_{eth} for selectivity towards ethenolysis, "mass bal." for mass balance. Internal standard: *trans*-decalin. The mass balance below 97% is primarily due to skeletal isomerization of **2** and **3**.

Exp. - #	Catalyst	Loading / ppm	T / °C	p / bar	Time / h	**2** / %	**3** / %	Conv. / %	S_{eth} / %	TON	Mass bal. / %
1597	**M1001**	"	"	10	"	55	56	74	77	11047	98
1601	"	"	80	"	"	56	58	75	79	11352	97
1604	"	"	60	20	"	71	72	85	89	14329	96
1607	"	"	"	30	"	78	80	87	92	15819	98
1610	"	"	"	40	"	83	86	91	94	16902	99
1613	"	"	"	50	"	84	87	91	97	17153	98
1619	"	"	"	40	1	81	83	88	96	16397	98
1598	**M1002**	50	60	10	"	52	53	59	94	10501	97
1602	"	"	80	"	"	49	50	62	93	9960	92
1605	"	"	60	20	"	62	64	69	96	12567	96
1608	"	"	"	30	"	66	69	72	99	13474	97
1611	"	"	"	40	"	68	70	73	99	13808	97
1615	"	"	"	30	1	70	67	74	99	13784	96
1599	**UltraCat**	"	"	10	0.5	56	57	79	74	11358	98
1603	"	"	80	"	"	55	57	80	74	11216	96
1606	"	"	60	20	"	70	72	87	85	14249	97
1609	"	"	"	30	"	74	76	87	88	14990	98
1612	"	"	"	40	"	76	78	87	89	15390	99
1616	"	"	"	30	1	78	81	90	90	15914	98

The activity of the CAAC-ligated catalysts towards ethenolysis of *trans*-alkenes was also investigated under optimal conditions for each catalyst. *trans*-alkenes are more difficult to transform via metathesis than *cis*-alkenes; at the same time, the product mixture of C18:1 isomerization is mainly comprised of more thermodynamically stable *trans*-C18:1 isomers. The results are summarized in Table S6.

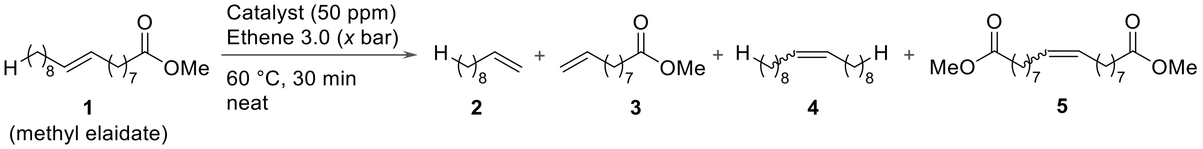

Table S6. Catalytic activity comparison of **M1001**, **M1002**, and **UltraCat** in ethenolysis of methyl elaidate. "Conv." stands for conversion, S_{eth} for selectivity towards ethenolysis, "mass bal." for mass balance. Internal standard: *trans*-decalin.

Exp. - #	Catalyst	p / bar	**2** / %	**3** / %	Conv. / %	S_{eth} / %	TON	Mass bal. / %
1625	**M1001**	40	59	61	64	96	11930	98
1626	**M1002**	30	29	30	31	98	5858	99
1627	**UltraCat**	30	44	44	53	84	8798	99

We also investigated the possibility to lower the catalyst loading for the ethenolysis reaction. As it is shown in Table S7, decreasing the catalyst loading of **M1001** to 25 ppm lowers the conversion to only 32%. Such dramatic decrease of the catalyst productivity is most probably due to the purity of the used ethylene. According to the product datasheet, ethylene 3.0 contains up to 15 ppmv oxygen that is possibly the most dangerous contaminant. In order to decrease the detrimental effect of ethylene contaminants, we added triethylaluminium to the reaction mixture.[5,6] As can be seen in Table S7, addition of 500–2000 ppm TEA indeed had a pronounced effect on the ethenolysis, increasing the overall conversion albeit decreasing the selectivity.

Table S7. Effect of triethylaluminium (TEA) addition on the MO ethenolysis outcome. “Conv.” stands for conversion, S_{eth} for selectivity towards ethenolysis, “mass bal.” for mass balance.

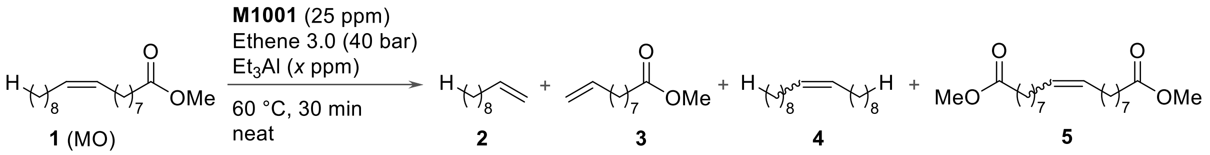

Entry	TEA / ppm	**2** / %	**3** / %	Conv. / %	S_{eth} / %	TON	Mass bal. / %
1644	-	30	32	32	98	12401	100
1725	500	43	43	44	94	17219	101
1728	1000	66	68	77	85	26756	101
1729	2000	57	59	59	99	23262	100

Overall, **M1001** turned out to be the best catalyst under our test conditions; it results in only slightly better conversions of methyl oleate than **UltraCat** but shows higher selectivities towards ethenolysis. Also, due to the lowest steric bulk, **M1001** affords the highest conversion of methyl elaidate which renders this catalyst the most promising towards ethenolysis of isomerized FAMEs. We considered the following conditions optimal: 50 ppm **M1001**, 60 °C, 40 bar ethylene, and 30 min reaction time.

6.3. Large-scale ethenolysis

Once the optimal ethenolysis conditions were established, we could ethenolyse the collective product of the long-term semi-RME isomerization. The ethenolysis was performed on a 250 mmol scale (Scheme S2) and afforded a product mixture that furnished a gas chromatogram shown in Figure S7. If compared with commercially available diesel (Figure S8), our product mixture effectively covers its entire boiling region, with minor differences such as a higher proportion of low-boiling compounds (retention time <10 min) and a lower proportion of compounds at retention times between 15–20 min. This would result in corresponding differences in the boiling point curve profile.

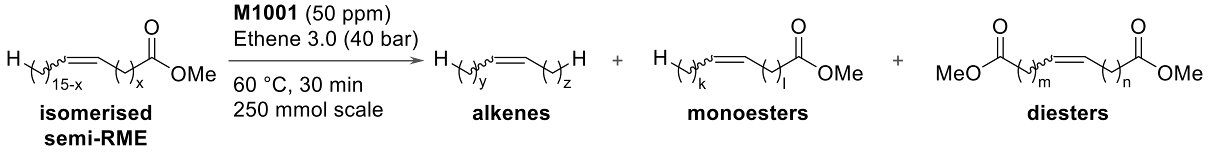

Scheme S2. Large-scale ethenolysis of isomerized semi-RME, with possible products.

Catalyst stock solution: a 10 mL oven-dried headspace vial was charged with the M1001 (8.6 mg, 1.1x scale) and transferred into a nitrogen-filled glovebox. In the glovebox, it was charged with 5.5 mL toluene.

Reaction procedure: in the glovebox, a steel autoclave (Figure S6) equipped with a Teflon-coated stir bar was charged with 112 mL isomerized semi-RME (250 mmol, 76% C18:1) and 5.0 mL catalyst stock solution (corresponds to 7.8 mg, 0.0125 mmol, or 50 ppm M1001), resulting in the toluene content of 4.3 vol-% in the mixture. The autoclave was sealed, taken out of the glovebox, pressurized with 40 bar ethylene 3.0 and placed in a preheated heating block. It was then stirred at 60 °C for 1 h, while ethylene pressure was maintained constant at 40 bar. Subsequently, the autoclave was cooled down to R.T., and the pressure was released. The reaction mixture was transferred into a 1 L beaker equipped with a stir bar, and 100 mL aq. hydrogen peroxide was added (35% v/v, technical purity) while cooling with a water/ice bath. The mixture was stirred for 30 min, the organic phase was separated, dried over $MgSO_4$ and decanted to afford 98.5 g of colorless liquid, which corresponds to 94% gravimetric recovery. The theoretical amount of the product mixture was calculated as a sum of the theoretical ethenolysis product amounts and the amount of C16:0 and C18:0 in the substrate. The residual amount of Ru was estimated to be below 3 ppm, as stated in the work of Knight et al.[7]

The density of the final mixture was measured upon weighing a 10.00 mL sample in a vial in 1.00 mL steps and was found to be 0.847 g/mL (Table S8).

Table S8. Density calculation of isomerized and ethenolysed semi-RME.

Volume / mL	Weight / g
1	0.85
2	1.694
3	2.54
4	3.387
5	4.236
6	5.083
7	5.93
8	6.779
9	7.625
10	8.469

Also, for comparison, the collective product of long-term isomerization of HOSOME was ethenolysed on a 300 mmol scale under the optimal conditions. The resulting gas chromatogram is shown in Figure S9.

As it can be seen, lower extent of double bond migration results in sharper distributions of alkenes and monoesters.

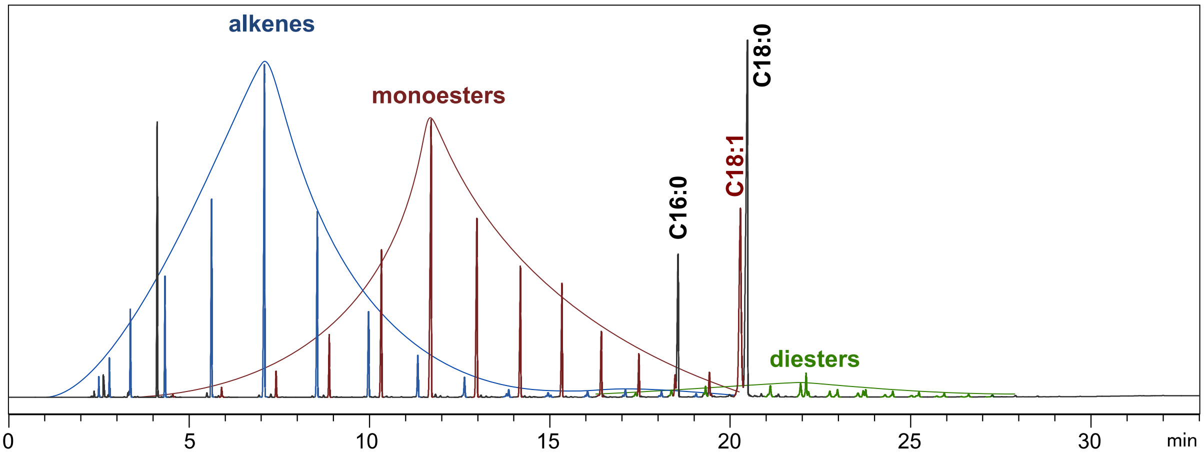

Figure S7. Gas chromatogram of isomerized and ethenolysed semi-RME (KOM-1659).

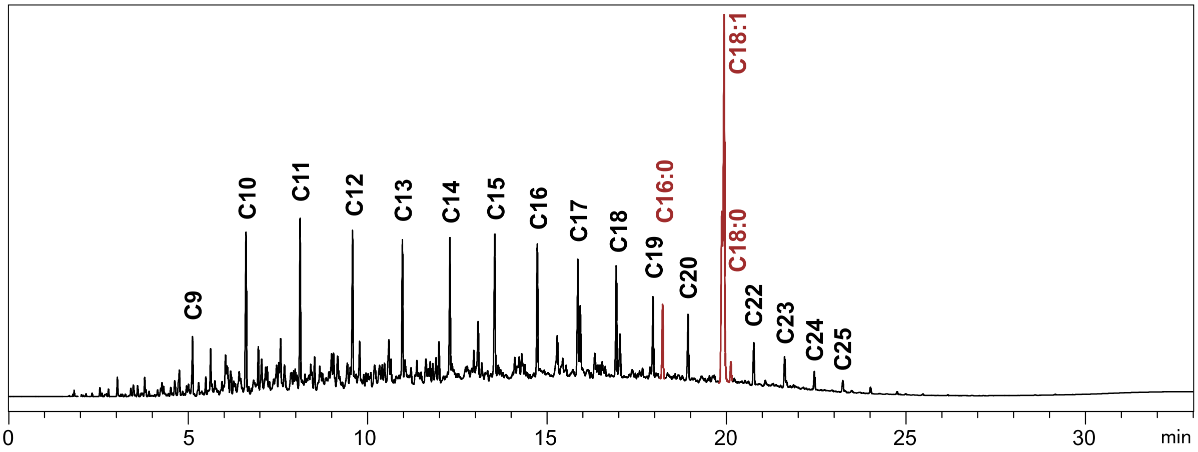

Figure S8. Gas chromatogram of commercial diesel, with biodiesel (present at 7 vol.-% as specified by EN 590) highlighted in red.

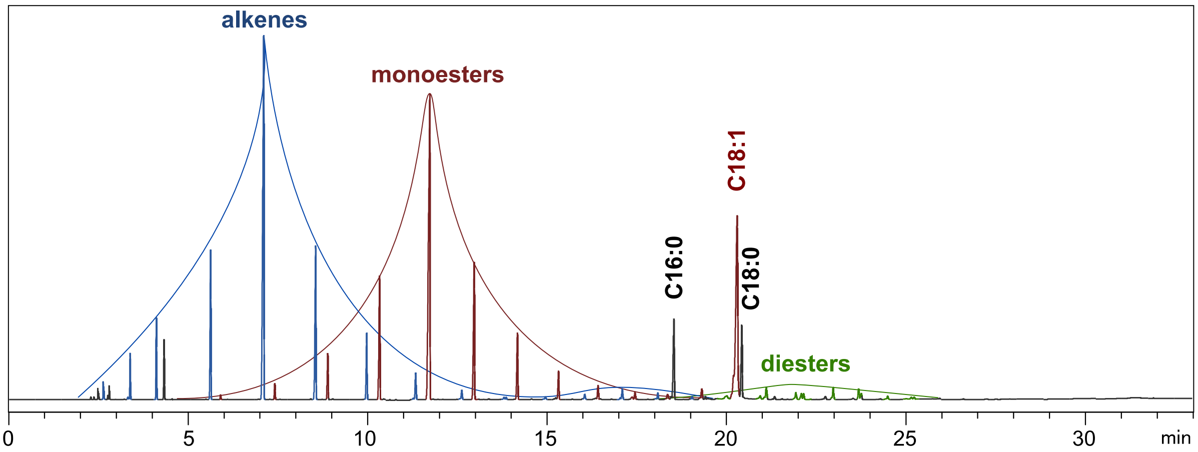

Figure S9. Gas chromatogram of isomerized and ethenolysed HOSOME (KOM-1577).

7. Distillation analysis

7.1. Experimental distillation analysis

According to EN 590, the boiling behavior of a fuel must be determined using experimental distillation according to EN ISO 3405 or ASTM D86. Both standards are generally equivalent and require a distillation apparatus that fulfills certain requirements specified in these standards. The boiling behavior of diesel fuel must fulfil the following rules:

1. <65% distilled at 250 °C.
2. >85% distilled at 350 °C.
3. >95% distilled at 360 °C.

In order to determine the boiling behavior of our envisioned biofuel, we required a distillation apparatus based on the EN ISO 3405 (ASTM D86) requirements. Since we did not have any more access to the Anton Paar apparatus used for our previous publication,[8] we designed our own system.

Our apparatus (Figure S10 and Figure S11) generally consisted of a commercially available 125 mL Engler flask, a thermocouple, a custom-made condenser, and a receiving cylinder. The Engler flask featured an NS 19/26 sleeve and all properties according to the standards (Figure S10,1). It was positioned inside a CNC-milled aluminium heating block (Figure S10, 2) with dimensions 85×85×15 mm featuring a concave round indentation in the middle (diameter of the sphere 70 mm, diameter of the cross-section with the surface 50 mm). The heating block was equipped with two heating rods (80 mm length, Ø 8 mm, 250 W) and a K-type thermocouple and was controlled by a simple temperature controller working in a full power mode during the measurement. The entire construction is surrounded by a CNC-milled chamotte insulating mantle (Figure S10, 3). The flask was surrounded by a metal enclosure shield made of an aluminium tube (150 mm long, Ø 80 mm) with a visor hole at a height of 30 mm from the bottom end and a cut-out to accommodate the distillation bridge (Figure S10, 4). The shield was placed on top of the heating block.

The temperature sensor (Figure S10, 5) was a Pt100 thermocouple (2×2 mm SMD chip) enclosed in a stainless-steel mantle (Ø 6 mm, wall thickness 1 mm) which is securely fastened inside a temperature sensor centering device (Bohlender® BOLA ground joint fitting connections NS 19/26, inner bore diameter 6 mm). The sensor was connected to ENDA ET2011 temperature controller that can interpret the input with 0.1 °C precision below 300 °C and 1 °C precision above 300 °C.

The condenser (Figure S10, 6) was custom-made by the faculty’s glassblower to fulfil the geometric requirements specified by the standards. The inner glass tube had a total length of 560 mm and an outer diameter of 14 mm and wall thickness of 1 mm. The upper end of the tube (30 mm) was fitted with a GL 18 connection (Figure S10, 7) that can securely hold the distillation bridge of the Engler flask; following 393 mm of the tube were surrounded by a Liebig condenser, and 135 mm was outside the cooling bath at the lower end, with the last 20 mm being bent to direct the flow into the receiving vessel. The bridge was positioned to have a decline of -15° to the horizontal axis. The Liebig condenser was filled with cold water and used without an active water flow during the measurement to avoid solidifying of the heavier distillation fractions inside the distillation bridge. The receiving vessel (Figure S10, 8) was a 100 mL laboratory measuring cylinder with 1 mL markings, ensuring ±0.05 mL precision.

The Pt100 thermocouple position was adjusted in a manner that the bottom of the steel mantle is 3 mm below the bottom part of the distillation bridge joint on the Engler flask. The correctness of the sensor position was confirmed by performing a test distillation with *n*-hexadecane, where the recorded T_{50} value was 280.8 °C (specified limits 272.2–283.1 °C for the manual distillation, true boiling point of *n*-hexadecane: 287.0 °C).

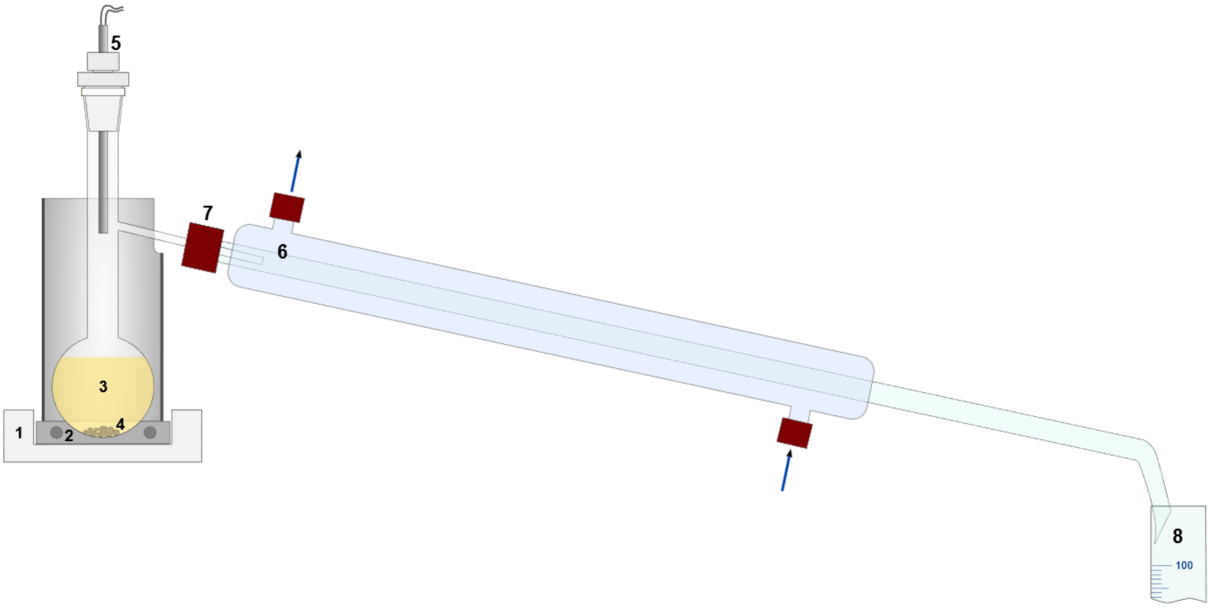

Figure S10. Schematic representation of the distillation analysis apparatus designed by us. 1) Chamotte insulation; 2) Heating block; 3) Engler distilling flask; 4) boiling stones; 5) Pt100 thermocouple and the temperature sensor centering device; 6) Condenser tube with a Liebig condenser; 7) GL18 fitting for connecting the distilling flask to the condenser tube; 8) 100 mL measuring cylinder.

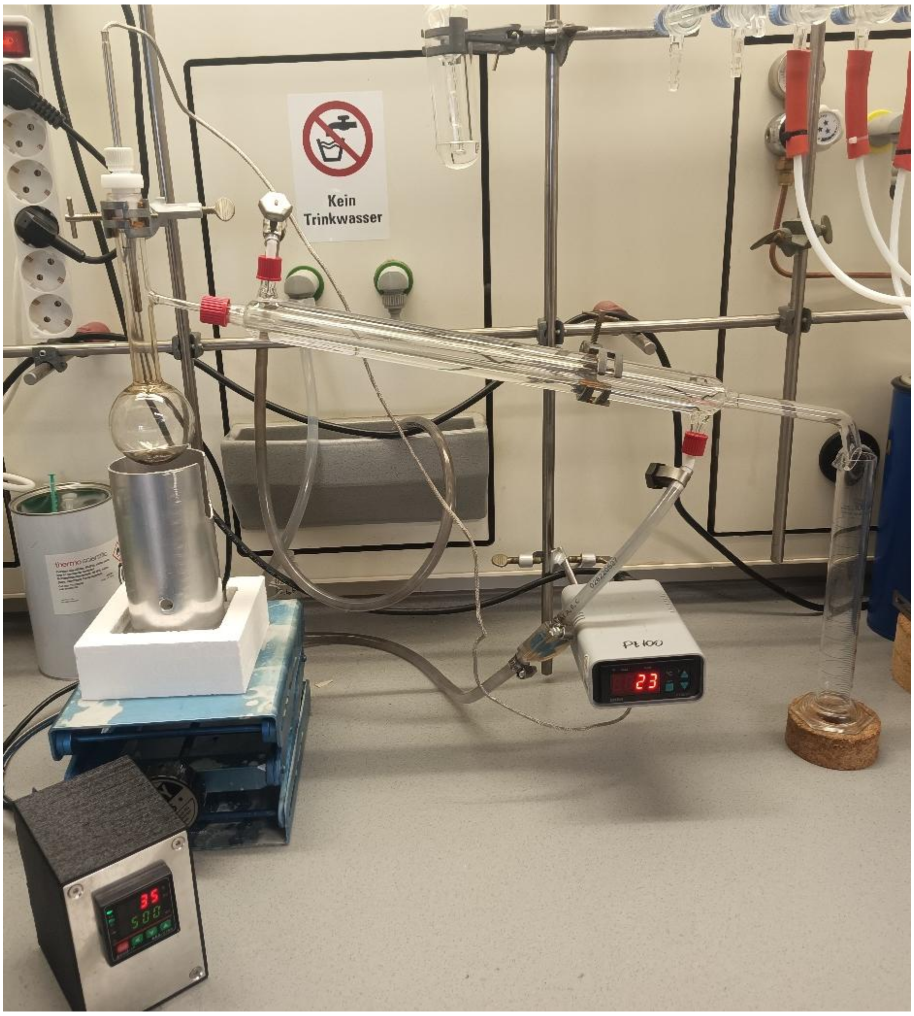

Figure S11. Photograph of the distillation apparatus.

The distillation was performed with a 100 mL sample volume in a manual mode; sample recovery was recorded visually in 5 vol.-% steps. The **initial boiling point (IBP)** was recorded, as soon as the first droplet fell down the receiving cylinder, and the **final boiling point (FBP)** was the highest head temperature achieved during the measurement and corresponds to 97–98% recovery. The temperature decrease after the FBP was referred to pyrolysis and the measurement was stopped.

With the distillation apparatus assembled and calibrated, we could perform distillation analysis of several reference mixtures and of the large-scale ethenolysis product mixtures (Table S9). We were happy to confirm that the recorded b.p. curve of commercial diesel has shared high similarity with the diesel b.p. curve reported with a licensed measuring device (Figure S12).[8] Most importantly, there was no discrepancy in the critical high-boiling region. The discrepancy in the low-boiling region was due to higher content of low-boiling alkanes in the new diesel sample compared to the old sample, as it was determined by GC-FID (Figure S14). The old sample, however, could not be used in this work since after 7 years of storage, separation of the fuel in two phases occurred, rendering it unsuitable for distillation analysis.

Table S9. EN ISO 3405 (ASTM D86) distillation data of several key mixtures and the large-scale ethenolysis mixtures. SM stands for self-metathesis, “isom.” for isomerization, “ethenol.” for ethenolysis.

Exp.- #	1576	1576	1662	1673	1577	1661	1674
Recovery / %	Diesel	Biodiesel	HOSOME ethenolysed	HOSOME, SM	HOSOME isom. + ethenol.	RME isom. + ethenol.	Jet A-1
IBP	137.2	317	79	282	93	80	129.1
5	171.1	333	137	311	138	124	160.4
10	189.7	336	154	320	157	145	170.0
15	199.7	337	164	324	168	161	175.8
20	209.2	338	172	326	176	173	180.6
25	220.1	339	179	328	183	188	184.4
30	229.4	340	187	330	191	202	187.7
35	237.8	340	194	331	198	216	190.9
40	246.7	341	200	333	206	226	194.7
45	255.7	341	207	336	214	236	197.8
50	265.5	341	215	338	221	250	200.9
55	275.2	342	221	340	229	264	204.6
60	283.5	342	228	343	236	281	207.9
65	292.2	343	233	346	246	302	211.5
70	301	343	238	348	259	317	216.9
75	311	344	245	351354	282	327	221.8
80	320	344	274	357	316	337	228.4
85	329	345	332	361	333	341	235.4
90	339	345	342	Cracking	340	345	245.6
93	348	346	350	-	343	349	253.7
95	354	346	354	-	347	350	262.3
FBP	357	Cracking	354	-	348	350	274.3

After we confirmed the accuracy of our apparatus, we could proceed with recording boiling point curves of experimental product mixtures. To our delight, the well-isomerized and subsequently ethenolysed semi-RME furnished a b.p. curve that is fully compliant with EN 590 requirements (Figure S13).

According to these results, if the FAME isomerization affords a product mixture with <34% non-migrated C18:1 isomers, the subsequent ethenolysis would result in an EN-590-compliant b.p. curve. As shown in Figure S13 for comparison, ethenolysis of poorly isomerized HOSOME (45% non-migrated isomer) furnishes a b.p. curve that is too light-boiling.

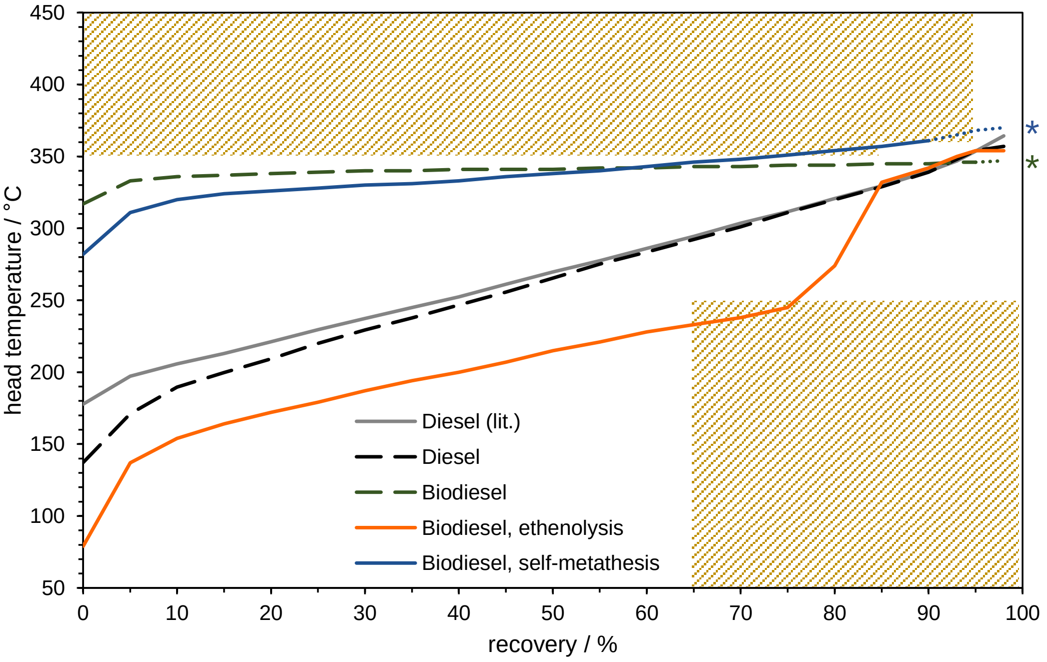

Figure S12. Experimental boiling point curves for diesel, biodiesel, as well as biodiesel modified via self-metathesis and ethenolysis. The dashed graph regions are the zones forbidden by the EN 590.

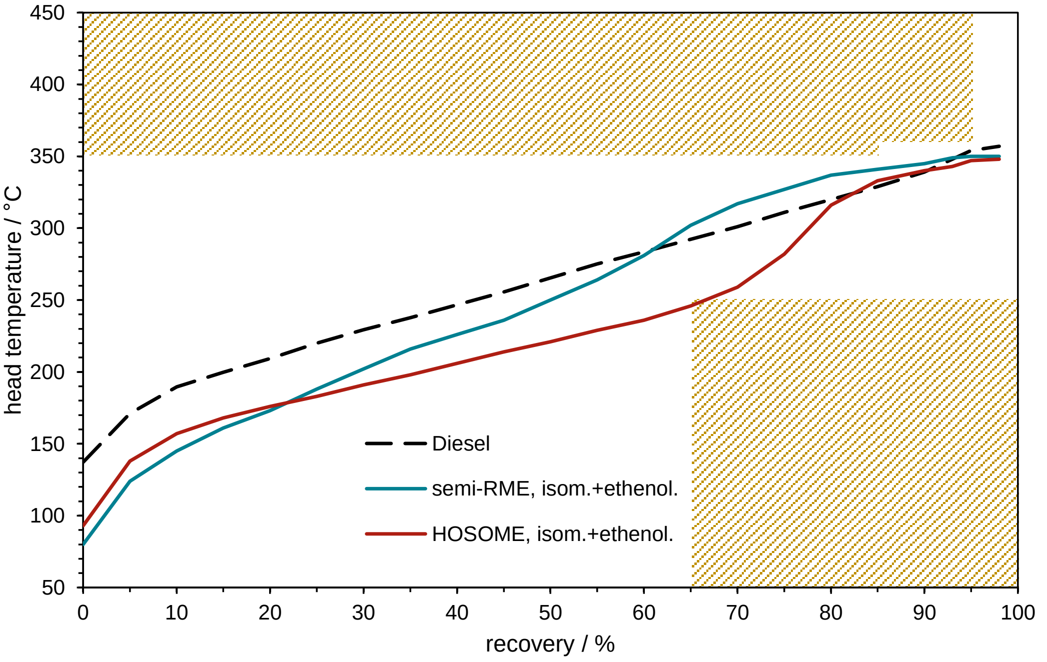

Figure S13. Experimental boiling point curves for isomerized and ethenolysed semi-RME and HOSOME. The dashed graph regions are the zones forbidden by the EN 590.

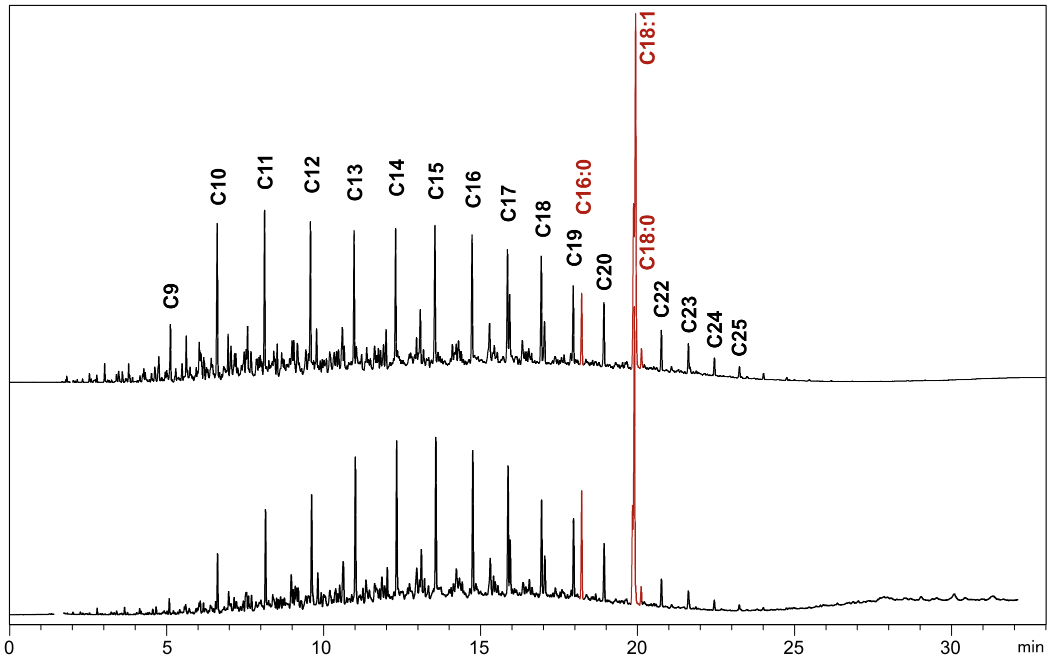

Figure S14. Comparison between the gas chromatograms of the new diesel batch and the old sample used in our previous work.[8] Raw data: KOM 1480, KOM 1576.

7.2. Cetane index

The **Cetane Index (CI)** was calculated by the formula according to ASTM D976:

$$\mathrm{CI} = 454{,}74 - (1641{,}416 \cdot \mathrm{d}) + (774{,}74 \cdot \mathrm{d}^2) - 0{,}554 \cdot \mathrm{T}_{50} + 97{,}803 \cdot (\log \mathrm{T}_{50})^2,$$

where d is the density (g·mL^{-1}) and T_{50} is the temperature value (in °C) of 50% recovery, as determined by ASTM D86.[9]

For isomerized/ethenolysed semi-RME, the CI value is 44.

7.3. Simulated distillation analysis

At the initial stages of this project, we entertained an idea of predicting a boiling point curve for every isomerization fraction shown in the main manuscript. For that, a simulated distillation software such as Envantage Dragon II SimDis can be used that estimates the b.p. curve of the analyte according to ASTM D2887. The D2887 distillation analysis results are not identical to the ASTM D86 (EN ISO 3405) results; therefore, they must be converted to D86-compliant data using the API correlation (provided within the text of ASTM D2887).The correlation formula is as follows:

$$t_n = a_0 + a_1 \cdot T_{n-1} + a_2 \cdot T_n + a_3 \cdot T_{n+1}$$

and the correlation coefficients are provided in the Table S10.

For simulated distillation analysis, HP-5 column with the following temperature program was used: Carrier gar N_2, split 100:1, constant flow 1.2 mL/min, injector temperature 255 °C, detector temperature 350 °C. Oven temperature was raised from 40 °C to 350 °C at a rate 10.0 °C/min and then held for 2 min. Chromatogram analysis was done using Envantage Dragon II SimDis software (30-day test version) with the D2887 method. The RT mix required for this was prepared from n-alkanes (C_5, C_6, C_7, C_8, C_{10}, C_{11}, C_{12}, C_{14}, C_{16}, C_{18}, C_{20}, C_{22}, C_{24}, C_{26}, C_{28}, ca. 100 mg each) dissolved in 10 mL CS_2, as specified by ASTM D2887.

Table S10. API correlation coefficients.

t_n / °C	a_0	a_1	a_2	a_3	T_{n-1} / °C	T_n / °C	T_{n+1} / °C
IBP	25.351	0.32216	0.71187	-0.04221	T_{IBP}	T_5	T_{10}
5%	18.822	0.06602	0.15803	0.77898	T_{IBP}	T_5	T_{10}
10%	15.173	0.20149	0.30606	0.48227	T_5	T_{10}	T_{20}
20%	13.141	0.22677	0.29042	0.46023	T_{10}	T_{20}	T_{30}
30%	5.7766	0.37218	0.30313	0.31118	T_{20}	T_{30}	T_{50}
50%	6.3753	0.07763	0.68984	0.18302	T_{30}	T_{50}	T_{70}
70%	-2.8437	0.16366	0.42102	0.38252	T_{50}	T_{70}	T_{80}
80%	-0.21536	0.25614	0.40925	0.27995	T_{70}	T_{80}	T_{90}
90%	0.09966	0.24335	0.32051	0.37357	T_{80}	T_{90}	T_{95}
95%	0.89880	-0.09790	1.03816	-0.00894	T_{90}	T_{95}	T_{FBP}
FBP	19.444	-0.38161	1.08571	0.17729	T_{90}	T_{95}	T_{FBP}

The HOSOME samples collected at all flowrates (as described in the main manuscript) were ethenolysed under optimal conditions and further analyzed using the Envantage Dragon II SimDis according to ASTM D2887 with the API correlation. We would expect that simulated results for flow rates of 0.5 mL/min and lower would furnish an EN 590-compliant b.p. curve while the results for the flowrates 0.6 mL/min and higher would protrude into the lower forbidden region of the distillation graph. Instead, all simulated b.p. curves were almost identical (Figure S15); it seemed that FAME isomerization at any flowrate would in the end bring the same result, which cannot be true.

Therefore, we grew suspicious and, once the experimental b.p. curves were determined, compared them with the simulated results. Indeed, as it is shown in Figure S16, the simulated curve of isomerized and ethenolysed semi-RME demonstrates an abnormal behavior and, what is more pronounced, the b.p. curve profile of ethenolysed biodiesel was significantly flattened (compare with Figure S12).

We suspect that such dramatic error is due to the ability of the API correlation to correctly resolve only complex refinery fractions of a symmetrical shape that exhibit a linear, evenly rising b.p. curves. Therefore, simulated predictions would only work well for such mixtures as diesel, kerosene, or gasoline. In case of mixtures consisting of sharp, baseline-separated peaks, the simulated boiling point curve is distorted. The distortion is the strongest in the region between 30 and 70% recovery since the correlation generates only three datapoints in that region (see Table S10). At the same time, the temperature at 65% recovery must be precisely known to estimate compliance with the EN 590. Also, peaks of very high intensity (e.g. methyl stearate or oleate) cause an abnormal shape of the curve.

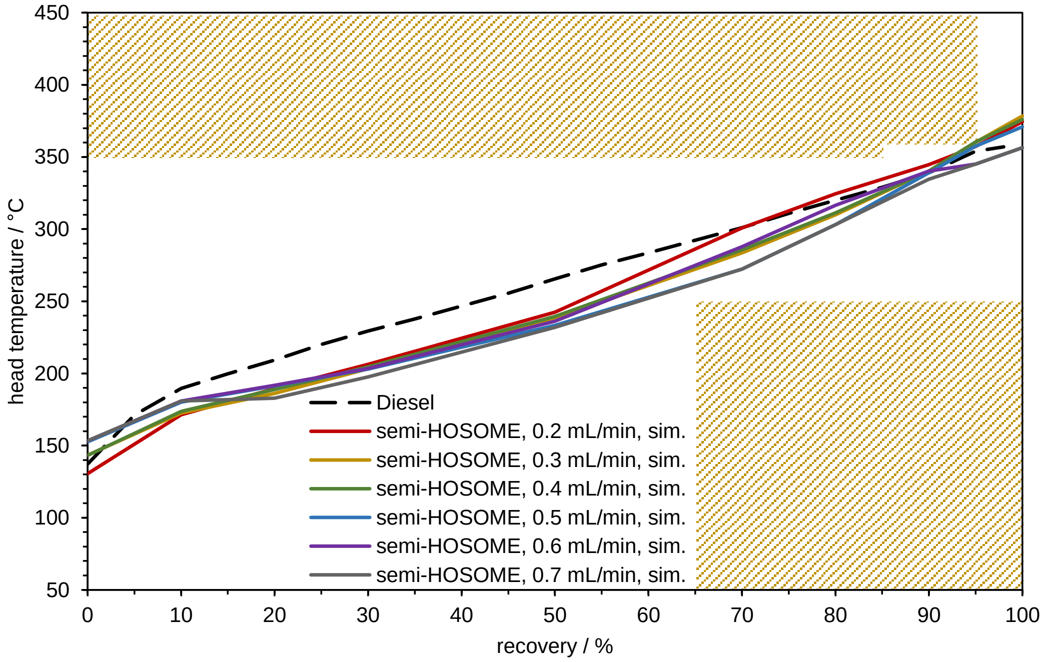

Figure S15. Simulated boiling point curves of semi-HOSOME which was isomerized at the specified flow rates (see Fig. 2 in the main manuscript) and subsequently ethenolysed. Obtained according to ASTM D2887+ API correlation. Raw data experiment numbers: KOM 1646–1650, 1660.

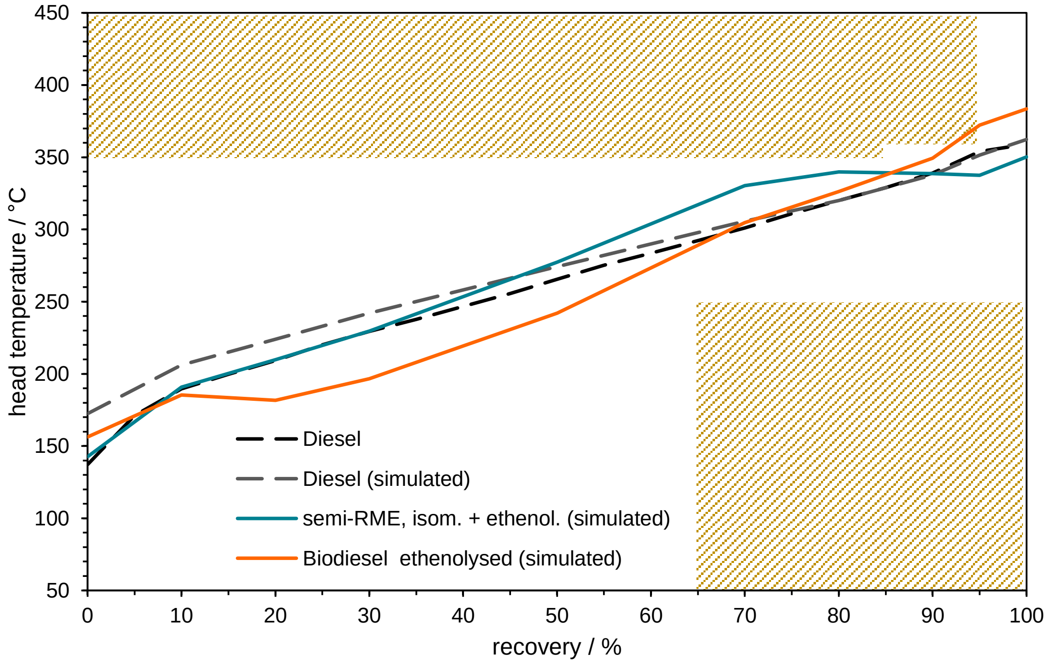

Figure S16. Simulated boiling point curves obtained according to ASTM D2887+ API correlation.

8. Attempts of subsequent hydrotreatment towards kerosene

8.1. Aviation kerosene specifications

After having established a synthetic pathway from RME to sustainable diesel fuel, we aimed to investigate a similar strategy towards aviation kerosene. The latter does not yet have a well-established sustainable production technology which is capable to substitute the fossil fuel to a significant extent; at the same time, passenger and cargo aviation runs exclusively on kerosene.[10]

Aviation kerosene is a narrow distillation fraction of crude oil (Figure S17) and is basically a part of diesel. In order to be used as a turbine fuel, kerosene must fulfil the requirements of ASTM D1655. This standard prescribes, among other parameters, a trace content of oxygenates and the following boiling behavior (Figure S18):

1. At least 10% recovery at 205 °C
2. Final boiling point at 300 °C
3. No more than 1.5% residue, no more than 1.5% loss

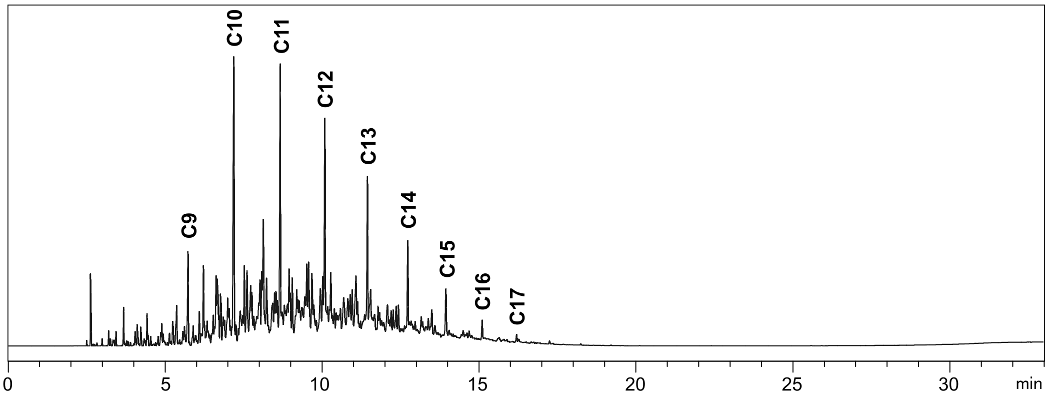

Figure S17. Gas chromatogram of commercial Jet A1 kerosene.

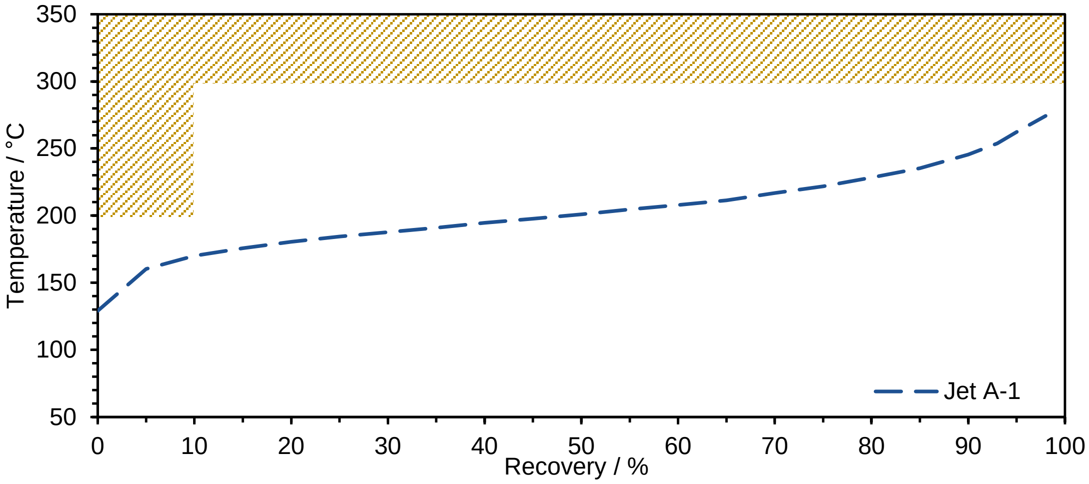

Figure S18. Experimental boiling point curve of Jet A1 kerosene. The curve is built using the values shown in Table S9. The dashed graph regions are the zones forbidden by ASTM D1655.

8.2. Experimental details

Aviation kerosene can be obtained from plant oils through hydrotreatment, which would naturally reduce the amount of oxygenates to trace levels.[11,12] We have rationalized that hydrotreatment of the sustainable diesel that we have obtained upon isomerization and ethenolysis of semi-RME, would effectively result in deoxygenation of all mono-and diesters, leaving behind only alkanes. As it can be

imagined when looking on Figure S7, after hydrotreatment only the blue product distribution pattern will remain; the latter features a distribution shape that is very similar to that of kerosene.

In order to test this hypothesis, we decided to perform hydrotreatment of semi-RME over a NiMo catalyst, which is the industrial standard.[11] However, NiMo catalysts are not available at general suppliers for academic use; therefore, we had to prepare such catalyst ourselves, according to the literature.[13] In the industry, the hydrotreatment is performed in a continuous-flow reactor; however, we did not possess the necessary equipment that would allow conducting a flow reaction at 100 bar H_2 and 300-350 °C. Therefore, we preferred small-scale, batch reactions in the autoclave.[14] Notably, the NiMo catalyst can only exhibit deoxygenating activity when sulphided; therefore, a correct amount of sulfur had to be added.

According to the literature,[14] a reaction time of 3 h after heating up the autoclave to 350 °C would be enough to reach equilibrium. We decided to consider the temperature 350 °C optimal but to reduce the reaction time to 2 h, since the heating up the autoclave took another 2 h. Thus, all additional time for set up and workup considered, we could perform two reactions per day (ca. 10 h in total).

Preparation of the NiMo/alumina catalyst

γ-Al_2O_3 (extrudates) was crushed and sieved. The fraction 0.25–0.50 mm was collected and dried at 120°C (ramp 2°C/min, hold 16 h). Then, 10.0 g of the sieve fraction was mixed with a saturated solution of nickel(II) nitrate (24.4 g, 82.2 mmol) and ammonium molybdate tetrahydrate (2.72 g, 2.20 mmol) via incipient wetness impregnation. Then, the sample was dried at 120 °C (ramp 2 °C/min, hold 16 h) and at 450 °C (ramp 2 °C/min, hold 4 h) to afford 21.3 g of the greyish-green product.

Reaction procedure

To prepare a 10x substrate:internal standard stock solution, 36.8 mL HOSOME (100 mmol, 92% C18:1, C18:2) and 5.00 mL tetradecane (19.1 mmol, 0.191 equiv.) were mixed in a vial.

A 50 mL Parr autoclave (Figure S19) equipped with a Teflon-coated stir bar was charged with NiMo on alumina (catalyst loading specified in), sulfur (catalyst:sulfur ratio specified in **Error! Reference source not found.**), and the 4.20 mL stock solution. The autoclave was sealed, pressurized with 100 bar hydrogen, and heated to 365°C (took ca. 2 h to heat up, upon reaching 365 °C, the pressure increased to 140 bar). Then, the reaction mixture was stirred at 365 °C for 2 h. Upon the reaction end, the autoclave was cooled down to r.t. and opened. Subsequently, one droplet of the reaction mixture was added onto 2 mL EtOAc and pushed through a short silica/$MgSO_4$ plug into a GC vial. GC-analysis was performed with GC-FID (GCC, BIOD).

For hydrotreatment of isomerized & ethenolysed RME, 10.0 mmol of this substrate was taken and no internal standard was used. The substrate was first hydrogenated over Pd/C (1 mol-% catalyst, 20 bar H_2, 80 °C, 30 min) to selectively remove double bonds that would result in unwanted side reactions during the hydrotreatment step. The hydrogenated substrate was filtered and further used as described above. Upon the reaction end, 0.5 mL of the reaction mixture was transferred into a GC vial and diluted with 1.0 mL CS_2. Then, a gas chromatogram for a simulated distillation analysis was recorded.

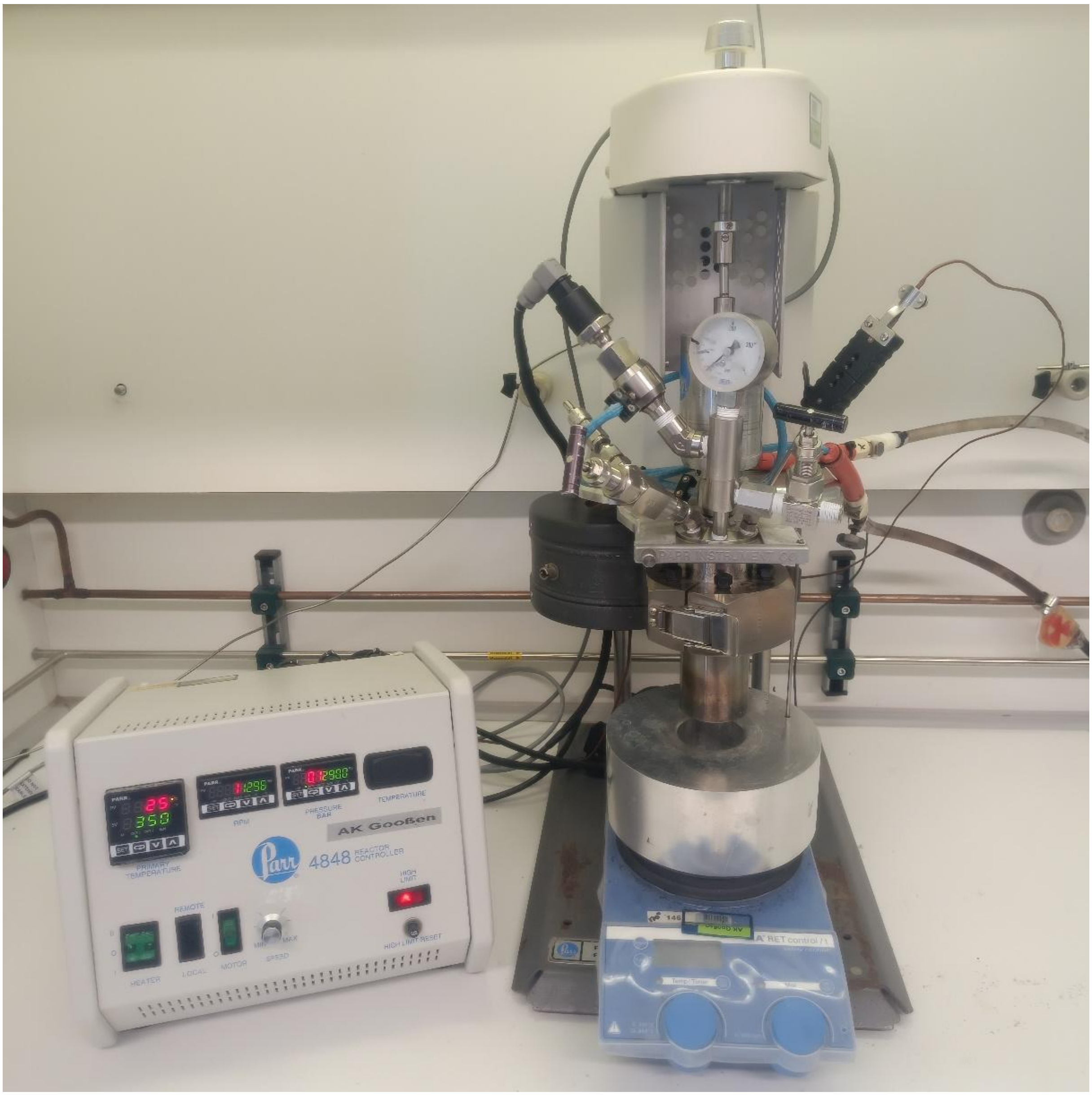

Figure S19. The 50 mL 316 steel Parr autoclave used for the hydrotreatment experiments.

8.3. Experimental results

The results of the deoxygenation reaction optimization, with the focus on maximum conversion, are summarized in Table S11. The reaction temperature was 365 °C since the heating process was naturally reaching this value despite any pre-adjustments of the heating program. The optimal conditions of HOSOME hydrotreatment (12 mol-% Mo, catalyst:sulfur wt.-ratio 4:1) allow for a quantitative conversion of methyl oleate and afford 57% octadecane **6**, 27% heptadecane **7**, and only several per cent of stearate and stearic acid.

In the next step, we applied these conditions onto the sustainable diesel fuel that we obtained from semi-RME. This has afforded a practically complete hydrogenation of oxygenates and resulted in a mixture shown in **Error! Reference source not found.**, bottom. Indeed, the signal profile in the chromatogram region of 3–13 min closely resembles the profile of Jet A1 kerosene. However, the region 13–22 min contains heavy-boiling components that are mainly the deoxygenation products of C16:0, C18:0, and C18:1 that were present in the substrate before the hydrotreatment. Removal of these esters

before hydrotreatment would thus result in sustainable, ASTM D1655-compliant kerosene. At the same time, distillation of the deoxygenation product mixture would afford kerosene and green diesel. However, the total energy input that is required to perform hydrotreatment and subsequent distillation will certainly render the final products non-economical.

Table S11. Optimization of hydrotreatment of HOSOME.

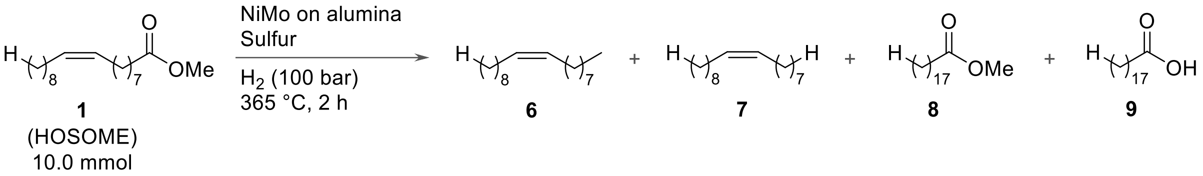

Exp.-#	Mo / mol-%	Cat. load. / mg	S / mg	Conv. / %	**6** / %	**7** / %	**8** / %	**9** / %
1682	6	204	-	>99	<1	<1	93	4
1679	"	"	102	78	18	6	32	10
1680	"	"	204	69	9	1	39	2
1681	"	"	51	97	25	22	27	9
1683	"	"	25	75	5	5	51	7
1684	12	408	102	>99	57	27	6	1

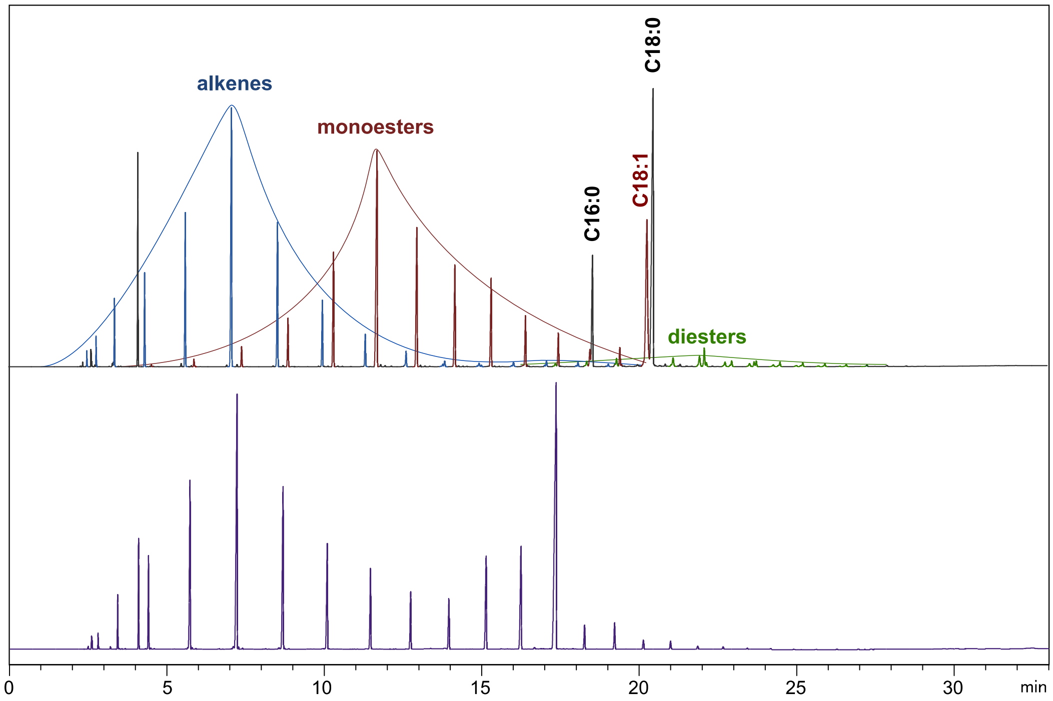

Figure S20. Top: gas chromatogram isomerized & ethenolysed semi RME. Bottom: gas chromatogram of the same mixture after hydrotreatment over a NiMo catalyst (KOM-1723).

9. References

[1] A. Behr, N. Döring, S. Durowicz-Heil, B. Ellenberg, C. Kozik, Ch. Lohr, H. Schmidke, *Lipid Fett* **1993**, *95*, 2–12.
[2] M. Kondratiuk, D. Gopinath, A. Elrrays, L. J. Gooßen, *Eur. J. Lipid Sci. Technol.* **2023**, *125*, 2200163.
[3] J. F. Goebel, F. Belitz, D. S. Prendes, Y. Haver, P. Diehl, M. Muhler, L. J. Gooßen, *ChemSusChem* **2024**, *17*, e202400094.
[4] A. B. Dixit, G. D. Yadav, *React. Funct. Polym.* **1996**, *31*, 237–250.
[5] K. M. Wampler, S. A. Cohen, G. E. Frater, L. Ondi, J. Varga, *Methods for Treating Substrates Prior to Metathesis Reactions, and Methods for Metathesizing Substrates*, **2016**, US9388097B2.
[6] K. M. Wampler, R. L. Pederson, *Synthesis of Pheromone Derivatives via Z-Selective Olefin Metathesis*, **2021**, WO2021247583A1.
[7] D. W. Knight, I. R. Morgan, A. J. Proctor, *Tetrahedron Lett.* **2010**, *51*, 638–640.
[8] K. F. Pfister, S. Baader, M. Baader, S. Berndt, L. J. Goossen, *Sci. Adv.* **2017**, *3*, e1602624.
[9] S. Bezergianni, A. Dimitriadis, *Renew. Sustain. Energy Rev.* **2013**, *21*, 110–116.
[10] International Energy Agency, *Energy Technology Perspectives 2020 – Analysis*, **2020**.
[11] R. K. Sharma, N. N. Bakhshi, *Can. J. Chem. Eng.* **1991**, *69*, 1071–1081.
[12] M. A. Rumizen, *Front. Energy Res.* **2021**, *9*, DOI 10.3389/fenrg.2021.760713.
[13] A. E. Coumans, E. J. M. Hensen, *Appl. Catal. B Environ.* **2017**, *201*, 290–301.
[14] R. Sotelo-Boyás, Y. Liu, T. Minowa, *Ind. Eng. Chem. Res.* **2011**, *50*, 2791–2799.
